# Integration of miRNA dynamics and drought tolerant QTLs in rice reveals the role of miR2919 in drought stress response

**DOI:** 10.1186/s12864-023-09609-6

**Published:** 2023-09-06

**Authors:** Deepesh Kumar, M. K. Ramkumar, Bipratip Dutta, Ajay Kumar, Rakesh Pandey, Pradeep Kumar Jain, Kishor Gaikwad, Dwijesh C. Mishra, K. K. Chaturvedi, Anil Rai, Amolkumar U. Solanke, Amitha Mithra Sevanthi

**Affiliations:** 1 ICAR-National Institute for Plant Biotechnology, New Delhi, 110012 India; 2 PG School, Indian Agricultural Research Institute, Pusa Campus New Delhi, New Delhi, 110012 India; 3 Division of Plant Physiology, ICAR-Indian Agricultural Research Institute, New Delhi, 110012 India; 4 ICAR-Indian Agricultural Statistics Research Institute, New Delhi, 110012 India

**Keywords:** Drought, miRNAs, Degradome, QTL, Thermal imaging, *Oryza sativa*

## Abstract

To combat drought stress in rice, a major threat to global food security, three major quantitative trait loci for ‘yield under drought stress’ (qDTYs) were successfully exploited in the last decade. However, their molecular basis still remains unknown. To understand the role of secondary regulation by miRNA in drought stress response and their relation, if any, with the three qDTYs, the miRNA dynamics under drought stress was studied at booting stage in two drought tolerant (Sahbaghi Dhan and Vandana) and one drought sensitive (IR 20) cultivars. In total, 53 known and 40 novel differentially expressed (DE) miRNAs were identified. The primary drought responsive miRNAs were Osa-MIR2919, Osa-MIR3979, Osa-MIR159f, Osa-MIR156k, Osa-MIR528, Osa-MIR530, Osa-MIR2091, Osa-MIR531a, Osa-MIR531b as well as three novel ones. Sixty-one target genes that corresponded to 11 known and 4 novel DE miRNAs were found to be co-localized with the three qDTYs, out of the 1746 target genes identified. We could validate miRNA-mRNA expression under drought for nine known and three novel miRNAs in eight different rice genotypes showing varying degree of tolerance. From our study, Osa-MIR2919, Osa-MIR3979, Osa-MIR528, Osa-MIR2091-5p and Chr01_11911S14Astr and their target genes LOC_Os01g72000, LOC_Os01g66890, LOC_Os01g57990, LOC_Os01g56780, LOC_Os01g72834, LOC_Os01g61880 and LOC_Os01g72780 were identified as the most promising candidates for drought tolerance at booting stage. Of these, Osa-MIR2919 with 19 target genes in the qDTYs is being reported for the first time. It acts as a negative regulator of drought stress tolerance by modulating the cytokinin and brassinosteroid signalling pathway.

**Supplementary Information:**

The online version contains supplementary material available at 10.1186/s12864-023-09609-6.

## Background

Rice (*Oryza sativa* L.) is the major food crop for more than half of the global population. The worldwide estimated production of milled rice in 2022–2023 is 512.4 million metric tonnes, the major portion of which comes from Asia [1]. Globally, around 164.19 ha are covered under paddy cultivation (USDA, 2020). As a result of climate change, drought and high temperatures have become more common in the last two decades. Among the various biotic and abiotic stresses that pose serious threat to sustainable rice production, drought remains the major constraint as it leads to substantial yield losses [2–4].

Plants have developed several mechanisms to cope with drought stress. They either escape drought stress by reducing their crop duration, especially by shortening the vegetative phase and tend to flower earlier, and thereby enter the reproductive stage before the onset of drought stress; or they avoid drought by maintaining higher tissue water potential. They also avoid drought through the acquisition of water by either developing a deeper root system or minimizing the rate of transpiration. The basic mechanisms behind drought tolerance include maintaining the turgor pressure by adjustment of the osmotic potential, increasing cell elasticity, reducing cell size, and acquiring desiccation tolerance via protoplasmic tolerance [5, 6]. Following this understanding, several attempts were made to develop transgenic lines by overexpressing some of the genes related to the physiological and biochemical basis of drought tolerance, viz., *D. stramonium adc* cDNA (polyamine biosynthetic pathway), *MnSOD*, late embryogenesis abundant (LEA) protein- *OsLEA3-1, OsDREB, OsNAC14, OsWRKY11* [7–12]. However, none of these led to a significant enhancement in drought stress tolerance that could be used at the commercial level [2, 13]. This reflected the fact that response and tolerance to stress mechanisms and their genetic control are complex, involving a multitude of genes, which necessitates a global understanding of drought stress tolerance, both at the genic and expression levels.

Drought stress at the reproductive stage is more detrimental to grain crops as it adversely impacts their grain yield. A few major quantitative trait loci (QTL) that govern yield under drought stress in field conditions, reported in the last decade, have been exploited in rice improvement in many Asian countries, through molecular breeding approaches [14, 15]. Muthu et al., [16] developed climate resilient BI (Backcross inbred) lines by pyramiding major QTLs for drought (qDTY1.1, qDTY2.1), salinity (Saltol), and submergence (Sub1) tolerance through marker assisted backcross breeding approach in the genetic background of a popular variety, White Ponni (IWP). Drought tolerant NI (near isogenic) lines, introgressed with two drought tolerant QTLs (*qDTY2.1* and *qDTY3.1)*, specific to reproductive stage, were developed in the high yielding elite cultivar, Pusa 44, through marker assisted backcross breeding [17]. In South East Asia, many abiotic stress tolerant varieties have been released for commercial cultivation, following marker-assisted pyramiding of QTLs for drought/salinity/submergence tolerance [18, 19]. Several QTLs have been identified for drought tolerance at vegetative stage and reproductive stage for different drought component traits including a few yield under drought QTLs. Of these three major QTLs (qDTY1.1, qDTY3.1 and qDTY12.1) have been identified with major effect on grain yield. qDTY1.1 was identified in F_3:4_ derived mapping population of N22 which is a drought tolerant *aus* cultivar with high yielding rice varieties such as IR64, Swarna and MTU1010 by Vikram et al., [20]. In addition to that, qDTY1.1 was also identified in the recombinant inbreed line (RIL) developed from drought tolerant donor parent Dhagaddeshi in the background of widely grown rice varieties Swarna and IR64 in the rainfed area which are high yielding but drought susceptible at reproductive stage [21]. Further, consistent effect of this QTL for grain yield under drought stress was observed by Vikram et al., [22] in a diverse panel of rice, elaborating the role of *sd1* locus under drought stress. qDTY3.1 was identified in the RILs of Swarna and Apo with a large effect on grain yield under drought stress condition using BSA approaches. Swarna is one of the major high yielding rice genotypes widely grown in the Asia and used in several breeding programmes intended for crop improvement but it has drought sensitive response at reproductive stage whereas Apo is aerobic-adapted variety with moderate tolerance to drought [23]. qDTY12.1 was reported to have large effect on grain yield under drought stress in the F_3_ derived line from Vandana and Way Rarem under reproductive stage [24]. Further Bernier et al., [25] confirmed the consistent effect of this QTLs under drought in diverse environments. Dixit et al., [26] confirmed the interaction of qDTYs 12.1 with qDTY_2.3_ and qDTY_3.2_ which enhanced the grain yield under drought stress condition. Though these yield under drought stress QTLs became a huge success at the field level, till date, none of the QTLs have been dissected to identify the underlying genes, despite the intense efforts taken towards fine mapping these QTLs [22, 27]. One of the reasons could be that regulatory elements such as microRNAs or small peptides might be playing the role of modulators of gene expression either at the post-transcriptional level, targeting mRNA for cleavage, or directing translational inhibition under drought stress.

MicroRNAs (miRNA) are endogenous single-stranded non-coding RNA molecules of approximately 22 nucleotides that bind to partially complementary sequences in their target mRNAs [28]. Plant miRNAs are derived from their RNA precursors by processing. Such precursors are occasionally transcribed from an intron or exon of a protein coding region, but most precursors are transcribed from the intergenic regions of genomes [29]. The role of miRNAs in many biological processes, such as development of roots, leaves, stems, and flower parts, is well established. miRNAs also play an important role in abiotic stress mitigation by modulating their abundance and controlling target mRNA or forming miRNA-protein complexes, which results in changes in the timing, transcript abundance, and tissue-specific expression of abiotic stress responsive mRNAs in tissues [30–32].

The first miRNA study on drought tolerance in rice reported that 11 families, including miR170, miR172, miR397, miR408, miR529, miR896, miR1030, miR1035, miR1050, miR1088, miR1126 were downregulated, while eight miRNAs, namely, miR395, miR474, miR845, miR851, miR854, miR901, miR903, miR1125 were upregulated under drought stress [33]. The miRNome of cv. N22 under drought stress at anthesis reported miRNAs within the QTLs regions related to drought tolerance traits (but not their target genes) besides stage- and variety-specific expression of miR164, miR396, miR812, and miR1881 [34]. Awasthi et al., identified four drought responsive novel miRNAs in cv. KMJ 1–12-3, viz., Osa-MIR12470, Osa-MIR12471, Osa-MIR12472, and Osa-MIR12473 at vegetative stage upon response to PEG induced drought. A recent report identified 109 miRNAs of which 58 families were differentially regulated under drought stress [35]. An overview of the available literature on drought responsive miRNAs is given in Supplementary Table 1.

In brief, the donor genotypes that have better drought stress tolerance, QTLs that confer sustainable yield under drought stress, and a large number of drought stress responsive genes as well as miRNAs are known in rice [36–38]. However, the molecular basis of QTLs in terms of drought stress tolerant genes and miRNAs is not yet known. In order to gain insight into the miRNAs that could be playing a role in drought stress tolerance, small RNA sequencing data for three rice varieties, namely, Vandana (VD; drought tolerant), IR 20 (drought sensitive), and Sahbhagi Dhan (SD; drought tolerant) were generated in the present study by imposing drought stress at the booting stage. The target genes of the drought stress responsive miRNAs identified from these three datasets were predicted using a variety of bioinformatics tools, besides nine independent degradome datasets. RiceMetaSys, a comprehensive drought stress responsive gene database [36], was used to validate the target mRNAs. Finally, we narrowed down the target genes of the drought responsive miRNAs in three major ‘yield under drought’ QTLs and validated the selected drought stress miRNAs and the predicted target genes by expression profiling in eight different rice genotypes of varying degrees of drought stress tolerance as well as bioinformatic analysis using the data available in the laboratory as well as the public domain.

## Results

### Small RNA-seq datasets

On an average, 10,631,187 (10.63 million) raw reads were generated from the flag leaves of cvs. VD, SD and IR20 per treatment. After rigorous pre-processing of the raw reads, on an average, 3.65 million (3,653,816) adaptor trimmed clean reads were obtained which were subsequently used for mapping against the rice genome reference. Average mapping percentage of the processed reads was 91.33%.

### Identification of known miRNAs in rice under well-watered control and drought stress conditions

**Fig. 1:**
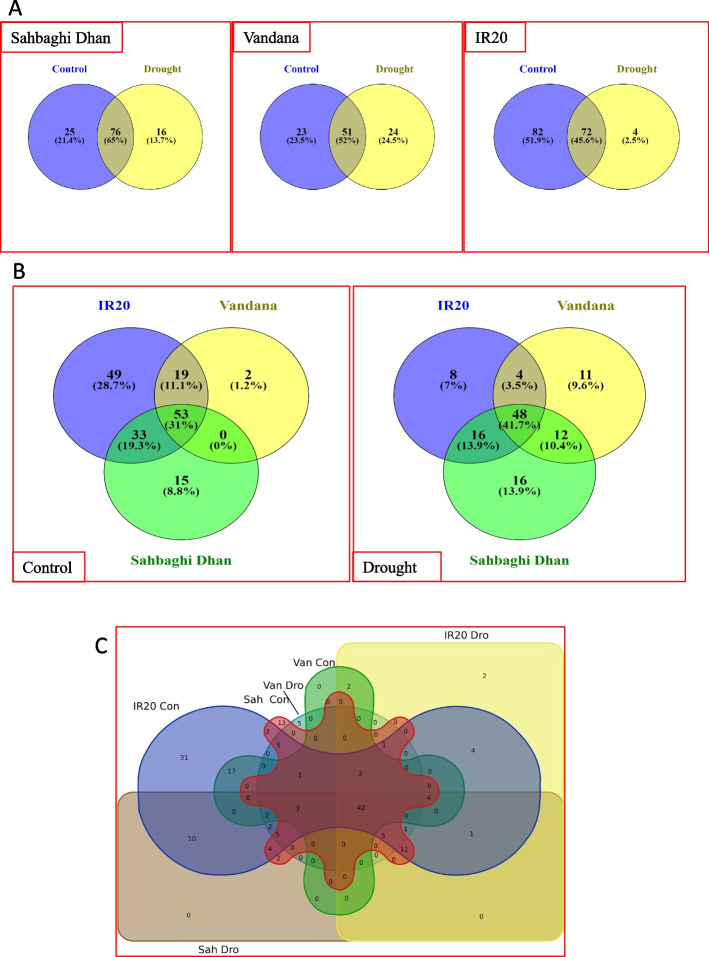
Multiple comparisons of known miRNAs identified under control and drought stress conditions in rice in three rice cultivars, Sahbhagi Dhan, Vandana, and IR 20 **A** Cultivar-specific comparison of miRNAs under well-irrigated and drought stress conditions at booting stage; **B** Treatment-specific comparison of miRNA identified in the three rice cultivars; **C** Multiple comparisons of known miRNAs among the three cultivars and two treatments

In order to comprehend the function of miRNAs under drought stress and their genotype specificity, we examined the expression of known miRNAs in three genotypes: Sahbaghi Dhan, Vandana, and IR20. Across the three rice genotypes, and two treatments (well-irrigated control and drought stress), a total of 178 known miRNAs were identified (Supplementary table 2A). Under the control conditions, 154, 101, and 74 known miRNAs were found in IR20, SD, and VD, which accounted for 171 unique miRNAs (Supplementary table 2A). The corresponding figure under drought stress was 115 with the maximum number of known miRNAs identified in SD (92) followed by IR20 (76) and VD (75) (Supplementary table 2A). IR20 had the least number of (4) drought stress specific miRNAs, accounting for just 2.5% (Fig. 1A). The number of known unique miRNAs specific to a genotype was 15 and 16 for SD, 2 and 11 for VD, and 49 and 8 for IR20, under control and drought conditions, respectively (Fig. 1B). While SD shared no common miRNAs with VD under control conditions, it shared 33 miRNAs with IR20. Between VD and IR20, 19 miRNAs were found to be common under control conditions. Interestingly, 53 common known miRNAs were detected in all three genotypes under control conditions while it was 48 miRNAs for drought stress conditions (Fig. 1B). From the multiple comparisons of miRNAs identified from the 3 genotypes and 2 treatments, we observed that 17 known miRNAs were common across all the genotypes and the two treatments, while 13, 31, 5 and 2 known mature miRNAs were unique to SD control, IR 20 control, VD drought and IR 20 drought respectively (Fig. 1C). Common miRNAs in all the three genotypes belonged to Osa-MIR159, Osa-MIR166, Osa-MIR396, Osa-MIR167, Osa-MIR156, Osa-MIR397, Osa-MIR398, Osa-MIR528 families (Supplementary Table 2A). Overall, the results showed that different sets of miRNAs were expressed in VD and SD under drought stress suggesting that the drought stress response mechanism was different in these two drought tolerant cultivars. For reliable identification of differentially expressed miRNAs, we applied read count filter (≥ 5 in both control and drought) after which 55, 76 and 48 known miRNAs in IR 20, SD and VD respectively were available for differential expression analysis between control and drought stress samples. The number of known differentially expressed miRNAs identified were 18, 23 and 12 of which 9, 18 and 6 were upregulated while 9, 5, and 6 were downregulated, in IR20, SD and VD, respectively (Supplementary Table 2B).

### Identification of novel miRNAs in rice under well-watered control and drought stress conditions

**Fig. 2:**
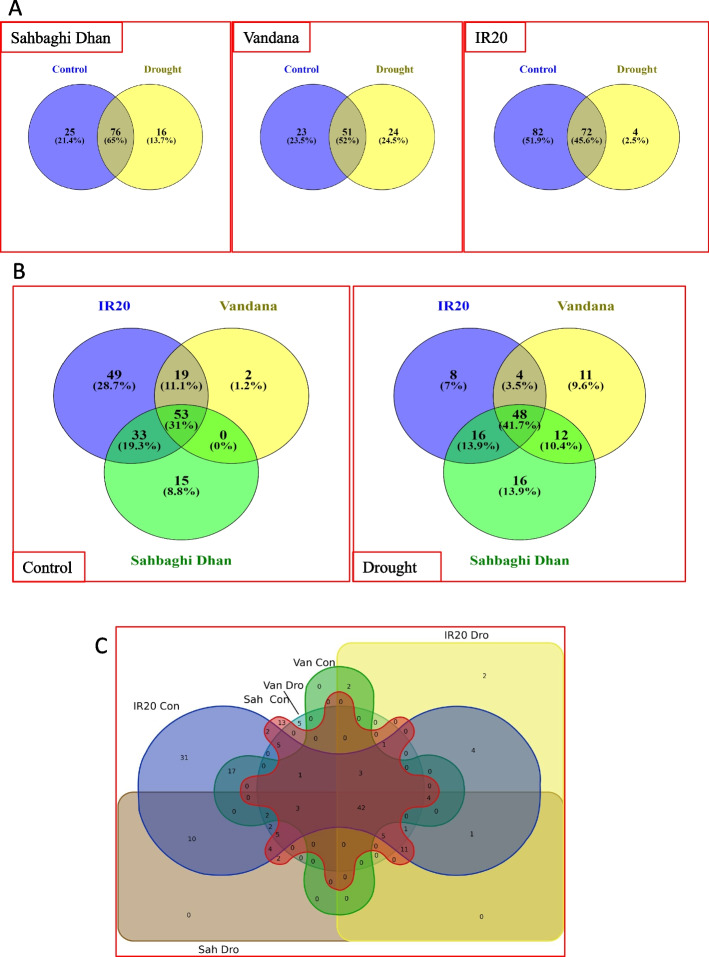
Multiple comparisons of novel miRNAs identified under control and drought stress conditions in rice in three rice cultivars, SD, VD and IR 20 **A** Cultivar-specific comparison of miRNAs under well-irrigated and drought stress conditions at booting stage; **B** Treatment-specific comparison of miRNA identified in the three rice cultivars; **C** Multiple comparisons of novel miRNAs among the three cultivars and two treatments

According to miRBase release 22.1 currently rice has 738 mature miRNAs with 604 precursors. Enormous increase in the advancement of sequencing technology and data generation emerged as a way to identify the novel miRNAs from different genotype to increase our understanding about role of miRNA and saturate the database. So, we explored the small RNA sequencing data to identify the putative novel miRNAs. The total number of putative novel mature miRNAs predicted by miRDeep2 under control conditions were 72, 77, and 38 in SD, IR20, and VD while under drought stress conditions, 57, 91 and 67 miRNAs were predicted (Supplementary Table 3A). Ninety-five novel miRNAs remained across samples and treatments after filtering (please see methods) (Supplementary Table 3B). Of these, 68 and 86 belonged to control and drought stress samples, respectively (Supplementary Table 3B). Between treatments, nine novel miRNAs were specific to control, while 24 were specific to drought stress (Supplementary Table 3B). In SD, IR 20, and VD, a total of 29, 30, and 9 novel miRNAs under control conditions and 42, 34, and 10 novel miRNAs under drought stress conditions were identified. Within genotype comparisons for the two treatments showed that 28, 23 and 7 novel mature miRNAs were common to both control and drought treatments, in SD, IR20 and VD, respectively (Fig. 2A). On the other hand, among the three genotypes, 1, 7, and 1 novel mature miRNA unique to control, and 14, 11, and 2 unique to drought stress were identified (Fig. 2A). Overall, fewer novel miRNAs were identified in VD in both the treatments. Further, 1, 20, and 20 novel mature miRNAs were unique to control, while 3, 30, and 20 were unique to drought stress in VD, SD, and IR 20 respectively (Fig. 2B). Four novel miRNAs were common to all the genotypes and treatments whereas 1, 7, 11, 9, and 2 were unique to SD control, IR20 Control, SD drought, IR20 drought, and VD drought (Fig. 2C). Differentially expressed novel miRNAs (log2FC > 1 or log2FC < -1) were 20 in IR20 of which 8 were downregulated while 12 were upregulated. In SD and VD, 14 and 6 novel differentially expressed miRNAs were identified of which 7, and 5 were downregulated, while 7, and 1 were upregulated (Supplementary Table 3C).

### Selection of drought stress specific miRNAs for target prediction

For target prediction, we selected 10 known and 9 novels differentially expressed miRNAs from the present study (based on higher read counts, lowest p value and log2FC > 1 or log2FC < -1), 8 previously identified miRNAs (based on miRNA microarray of IR64 and N22 control and drought stress samples) from our lab [39, 40], and two known miRNAs on the basis of differential expression under drought from [41]. Known miRNAs selected for target prediction included Osa-MIR166j-5p, Osa-MIR5079a, Osa-MIR168a-5p, Osa-MIR167h-3p, Osa-MIR159f, Osa-MIR528-5p, Osa-MIR531a, Osa-MIR531b, Osa-MIR530-5p, Osa-MIR408-3p, Osa-MIR820a, Osa-MIR5791, Osa-MIR156k, Osa-MIR2091-5p, Osa-MIR2091-3p, Osa-MIR2919, Osa-MIR396a-3p, Osa-MIR3979-3p, Osa-MIR399j, and Osa-MIR5072. Novel miRNAs selected were chr10_27362IR209A, chr08_25001IR209A, chr01_4341IR20S10A, chr01_6028IR20S10A, chr02_20631IR20S9B, chr12_38018S7AVan, chr04_25735S14AStr, chr11_38583S14AStr, and chr01_11911S14AStr. Secondary structure of the selected novel miRNAs is given in Supplementary Fig. 3. Out of the nine novel miRNAs selected for target prediction, three were unique to SD (chr04_25735S14AStr, chr11_38583S14AStr, chr01_11911S14AStr), two were unique to IR 20 (chr01_4341IR20S10A, chr10_27362IR209A), one was common to IR20 and SD with contrasting differential expression (chr08_25001IR209A), one was common to VD and IR 20 with contrasting differential expression (chr12_38018S7AVan), while, two were common to all the three genotypes (chr01_6028IR20S10A, chr02_20631IR20S9B), of which one was differentially expressed in cv. IR 20.

### Target prediction of the differentially expressed miRNAs identified

**Fig. 3:**
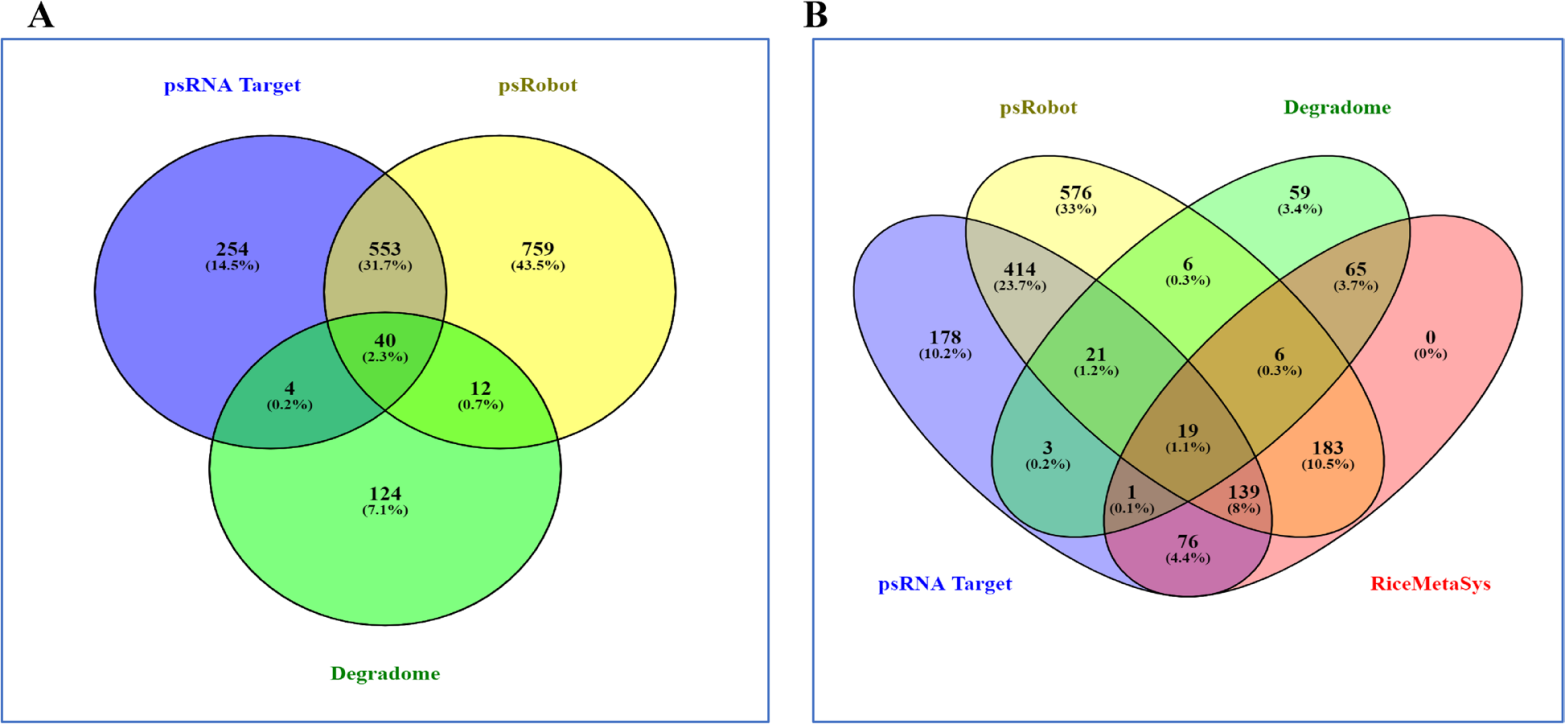
Multiple comparisons of the target genes of the differentially expressed miRNAs identified by different tools. **A** Target genes identified by psRNA Target, psRobot software and degradome datasets **B** Comparison of the target genes identified by the three tools with a database on drought stress responsive genes in rice, RiceMetaSys

To increase the reproducibility of predicted target genes and overcome the limitation of the available tools, we utilized the three different algorithms or tools for identification of target genes viz psRobot, psRNA Target and Cleaveland pipeline. psRNA Target and psRobot software predicted 851 (Supplementary Table 4A) and 1364 (Supplementary Table 4B) target genes for the 29 selected miRNAs, but the combined degradome analysis of nine different libraries gave only 180 (Supplementary Table 4C) target genes (of category 0–2) (Fig. 3A). Overall, a total of 1746 unique target genes were predicted by at least one tool (Supplementary Table 4D). However, only 40 target genes were common to all the three target prediction tools while, 759, 254 and 124 target genes were exclusively identified by psRobot, psRNA Target, and degradome analysis, respectively (Fig. 3A). While psRNA and psRobot shared 36.8% of the predicted targets, they had poor support from the degradome analysis. To further test the drought responsive nature of the predicted target genes, they were searched in RiceMetaSys, (a comprehensive drought stress responsive database developed from the meta-analysis of transcriptome data). RiceMetaSys showed that 28% of the target genes (489 out of 1746) were drought responsive genes in at least one microarray dataset (Fig. 3B and Supplementary Table 4E). Among the tools used, 91 (50.56%) genes from degradome analysis, 235 (27.61%) from psRNA Target and 347 (25.44%) genes from psRobot tool were supported by RiceMetaSys (Fig. 3B). Thus, though the targets predicted by degradome analysis were least common with those predicted by the psRNA and psRobot tools, the former had the highest support from experimental data under drought stress. Multiple comparisons of the identified target genes with RiceMetaSys further showed that 139 genes between psRobot and psRNA Target, six genes between psRobot and degradome, and 19 genes from all the three tools were common, and thus had ample support for their drought stress responsive nature (Fig. 3B). In the degradome analysis, 57 out of 180 target genes were detected in more than one library while 20 were supported by more than five libraries (Supplementary Table 4F). These 20 genes corresponded to eight of the differentially expressed miRNAs, including Osa-MIR156k, Osa-MIR159f, Osa-MIR2919, Osa-MIR396a-3p, Osa-MIR408-3p, Osa-MIR528-5p, Osa-MIR168a-5p, Osa-MIR531b. Some of the major target genes were, LOC_Os01g59660 (MYB family transcription factor, putative, expressed), LOC_Os02g04680 (OsSPL3—SBP-box gene family member, expressed), LOC_Os06g45310 (OsSPL11—SBP-box gene family member, expressed), LOC_Os01g69830 (OsSPL2—SBP-box gene family member, expressed), LOC_Os08g37670 (plastocyanin-like domain containing protein, putative, expressed), LOC_Os06g37150 (L-ascorbate oxidase precursor, putative, expressed), LOC_Os02g58490 and LOC_Os04g47870 (PINHEAD, putative, expressed) (Supplementary Table 4F). Notably, most of these genes belonged to SPL, SBP-box gene family.

### Co-localization of target genes of drought responsive miRNAs in major QTLs for yield under drought stress

**Fig. 4:**
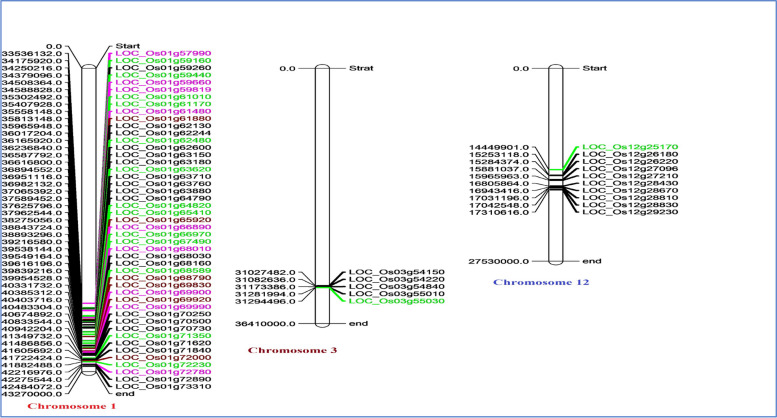
Target genes (of the differentially expressed miRNAs identified under drought stress) present in the three known major QTLs (qDTY1.1, qDTY3.1 and qDTY12.1) for yield under drought stress region (black), genes supported by RiceMetaSys (green), genes validated by expression analysis (purple), and genes common to RiceMetaSys and expression analysis (dark brown)

**Fig. 5:**
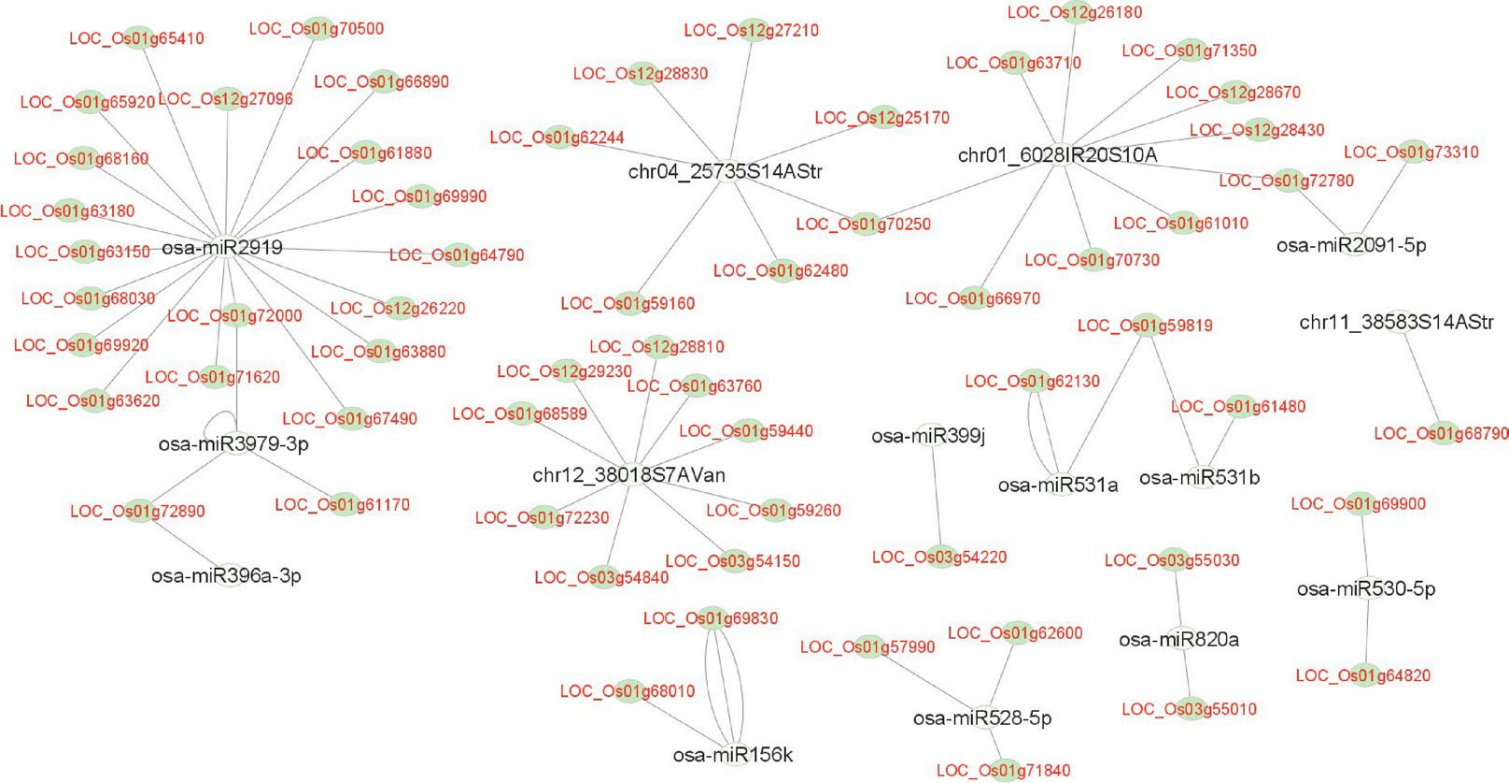
Interaction of the QTL-specific target genes of the differentially expressed miRNAs as shown by Cytoscape_3.9.0

Several attempts have been made to fine map the major QTLs under drought stress for identification of underlaying genes in rice but very little success is achieved. Here we have exploited the multiomics approaches by integrating the miRNA-mRNA knowledge for the first time to identify the genes governing the tolerance/susceptible response of rice underlying the major QTLs. The rice microsatellite marker intervals, namely, RM212-RM12233 (33,053,493- 42,415,653 bp), RM520-RM416 (30,912,691–31,248,603 bp) and RM28048-RM512 (14,106,460- 17,395,485 bp), spanning the major drought stress QTLs, qDTY1.1, qDTY3.1 and qDTY12.1, respectively, were retrieved. Out of the 1746 unique target genes identified, 61 genes pertaining to four novel and 11 known miRNAs were found to be localized within the drought QTL regions genes (Fig. 4 and Supplementary Table 5). Of these, the highest number of genes were localized in the qDTY1.1 (46 genes), followed by qDTY12.1 (10 genes) and qDTY3.1 (5 genes). From the network analysis, it became evident that Osa-MIR2919 had the highest number of target genes (19) in the QTL regions while single target genes were found for chr11_38583S14AStr, Osa-MIR159f, Osa-MIR396a-3p and Osa-MIR399j. Common target genes for more than one miRNA included LOC_Os01g72890/Osa-MIR396a-3p/Osa-MIR3979, LOC_Os01g72000/Osa-MIR3979/Osa-MIR2919, LOC_Os01g72780/Osa-MIR2091-5p/chr04_25735S14A, LOC_Os01g70250/ chr04_25735S14AStr/ chr01_6028IR20S10A and LOC_Os01g59819/Osa-MIR531a and b (Fig. 5). Out of the 61 target genes, 22 genes matched with the drought responsive genes of the RiceMetaSys database.

### GO enrichment and pathway analysis

**Fig. 6:**
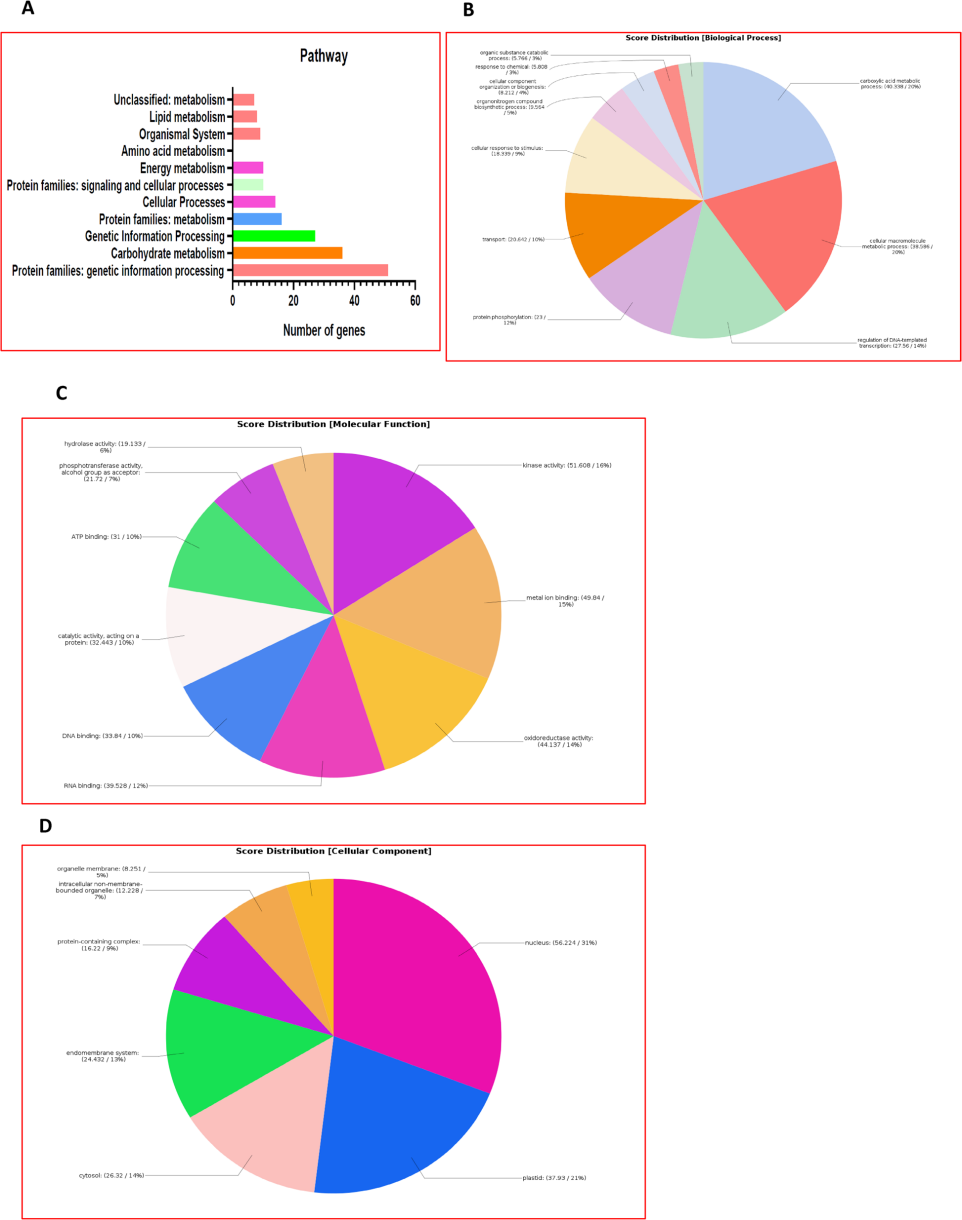
Go enrichment and Pathway analysis of the target genes specific to drought stress (as identified from the inventory, RiceMetaSys) **A** Target genes of the drought responsive miRNAs involved in various pathways; **B** Target genes of miRNAs enriched in biological processes; **C** Target genes of miRNAs enriched in molecular functions; **D** Target genes of miRNAs enriched in cellular components

We selected only those 489 target genes that matched with the drought responsive genes from the RiceMetaSys database and used the same as input for Blast2go and KEGG analysis. In KEGG pathway analysis, 230 (47%) of the 489 genes were found to be represented in different pathways of which metabolism of the major biomolecules and compound binding were found to be the prominent ones (Fig. 6A). Highest number of genes were involved in the carbohydrate metabolism (36 genes) followed by genetic information (replication, transcription, translation, folding, sorting and degradation of proteins etc.), cellular processes (14 genes), energy metabolism, amino acid metabolism, lipid metabolism and environmental information processing etc. (Fig. 6A). Out of the 489 genes examined, 424 could be found in the blast2go database, of which, 259 genes were found to be involved in various biological, molecular and cellular process. Of these, 232 genes were involved in biological process while 175 and 108 genes respectively belonged to molecular function and cellular component. The number of unique gene was the maximum (52 genes) in biological process. Biological process was most enriched in carboxylic acid metabolic process (40.38%), followed by, cellular macromolecule processes, regulation of DNA-template transcription and protein phosphorylation (Fig. 6B). Among the genes for cellular components, most of the genes were in the nucleus (56.224%), followed by, plastids and cytosol (Fig. 6C), whereas in molecular function category, genes were involved in kinase activity (51.60%), and metal ion binding (Fig. 6D).

### Sequence variation, promoter analysis and protein modelling

Since the role of Osa-MIR2919 under drought stress in rice was recognized for the first time and it singly accounted for 31.15% (19/61) of the target genes in the QTL, it was analyzed further. Sequence analysis of precursor and promoter of Osa-MIR2919 revealed that while precursor sequence was conserved in N22 and IR64, the promoter region harboured 2 SNPs (Supplementary Table 6A). The major cis elements were AACA (TAACAAACTCCA) motif involved in endosperm-specific negative expression of the gene, at 1157 bp position and GARE-motif (TCTGTTG), a gibberellin-responsive element, at 1145 bp position for the Osa-MIR2919 and these were not altered by the two SNPs.

**Fig. 7:**
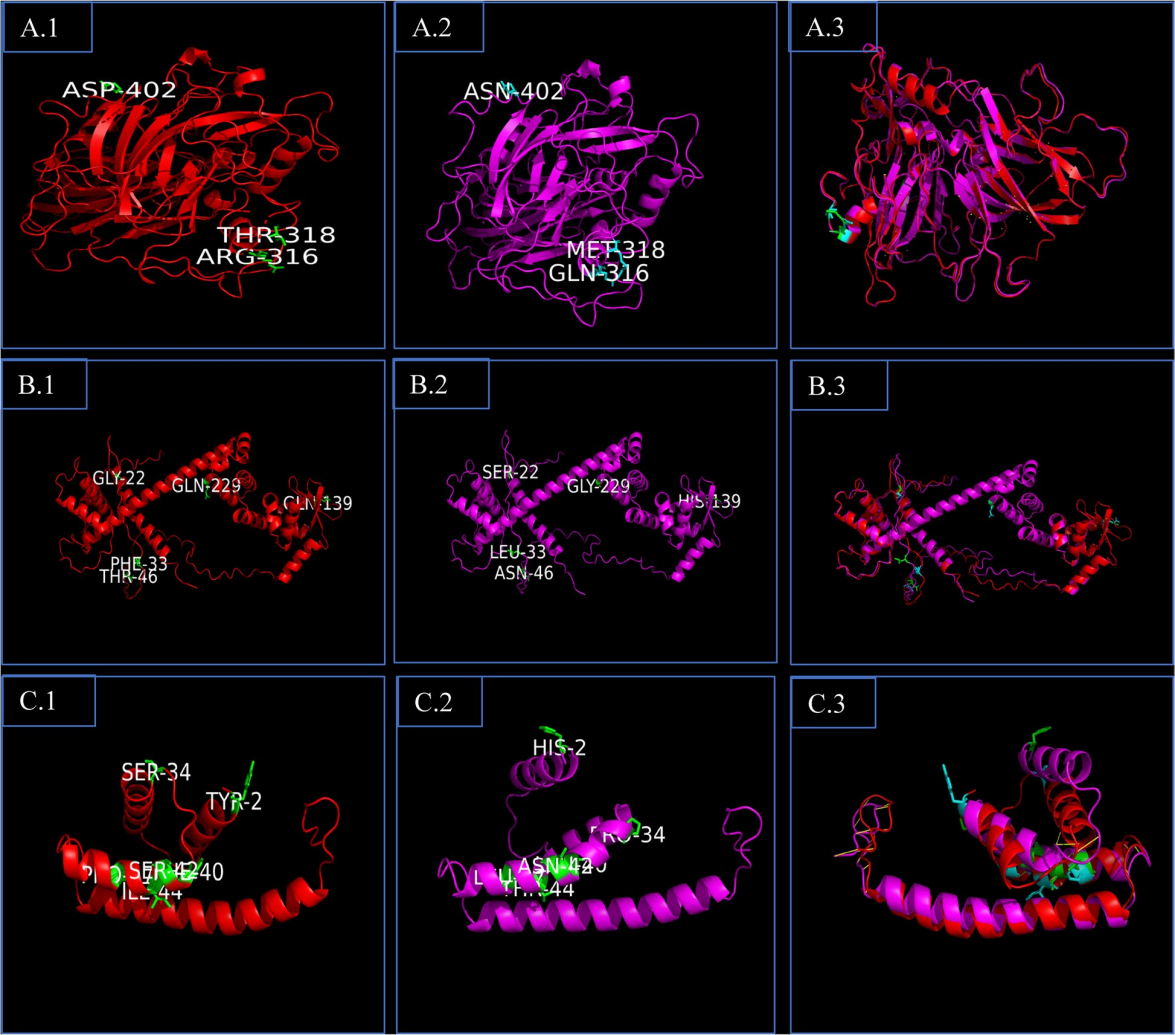
3D model of Proteins; **A** LOC_Os01g63180; **B** LOC_Os01g66890; **C** LOC_Os01g71620; Left panel (N22), middle panel (IR64) and right panel (Superimposed image)

Ten out of these 19 target genes showed amino acid (aa) substitutions between of N22 and IR64 (Supplementary Table 6B). Of these 10 genes, five had more than 2 aa substitutions. We could subject only three of them for protein modelling while the structure of other two genes could not be taken up due to sequence limit of > 1500. The multiple alignment of LOC_Os01g66890 showed five amino acid substitutions and multiple changes in the secondary structure as well (Supplementary Fig. 2 and Supplementary Table 6B). However, 3D structures did not show substantial differences as the RMSD value was only 0.304 (Fig. 7B). Six aa changes in LOC_Os01g71620 led to changes in both the secondary and tertiary structures between N22 and IR64 (Supplementary Figs. 2, 6B, and Fig. 7C). Superimposition of the two structures gave a very high RMSD value of 2.002. In the case of LOC_Os01g63180 (laccase-6 precursor), 3 aa changes led to multiple changes in the number and the length of the helices between N22 and IR64 alleles though their superimposition of 3D structures gave a smaller RMSD value of 0.141 (Supplementary Figs. 2, 6B, and Fig. 7A). Since the secondary structures are responsible for the local stability of proteins, the differences observed between IR64 and N22 in all the three genes could have a role on their functionality under stress.

### Physiological and biochemical response of the rice genotypes to drought stress at booting stage

**Fig. 8:**
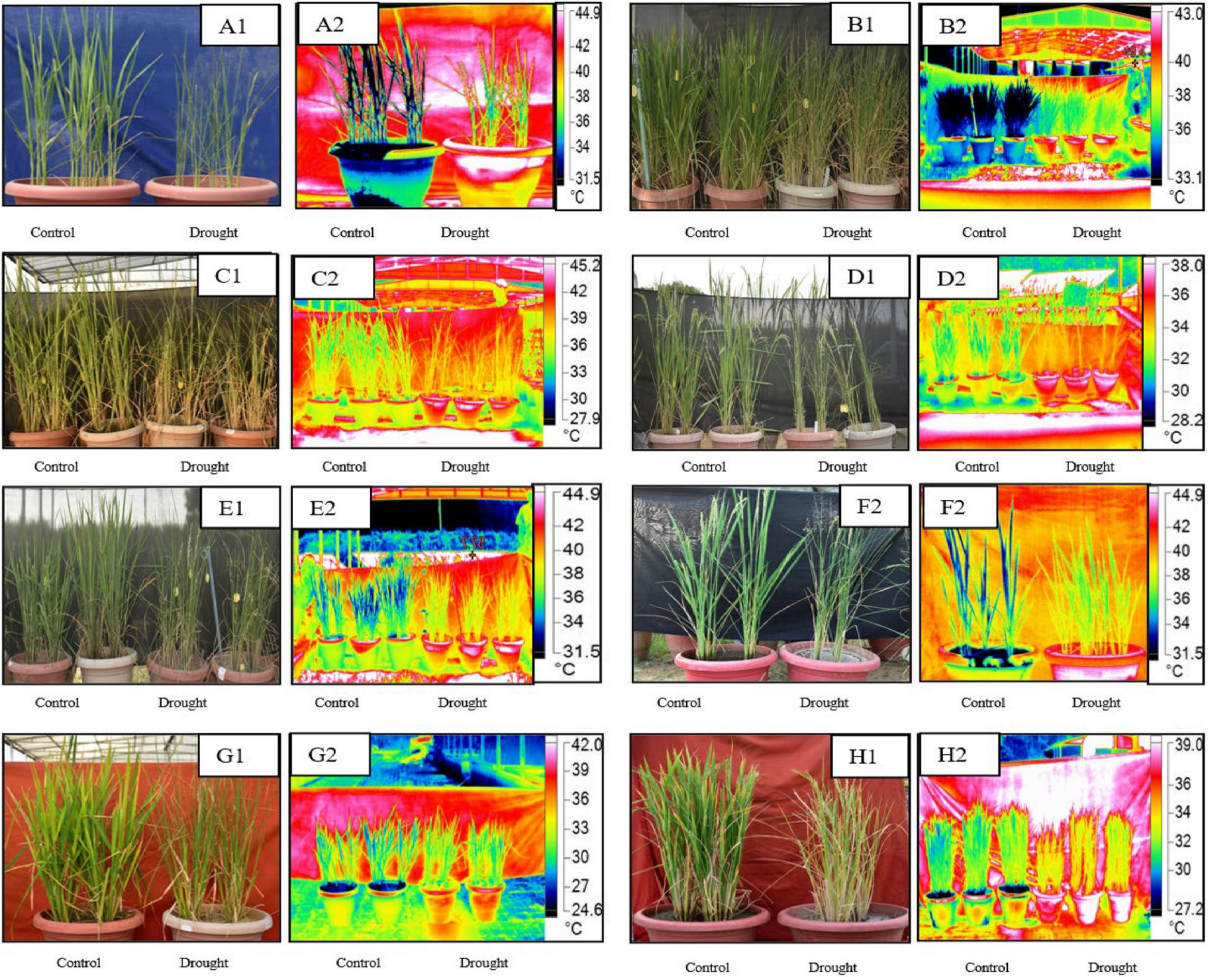
Visual and thermal imaging (Fluke TiX620 Infrared Camera): Thermal image of **A** KT, **B** IR 64, **C** N22, **D** RS, **E** VD, **F** TP, **G** SD and **H** DRR Dhan under well-watered (left panel) and drought stress (right panel) conditions

As every genotype may have a different biochemical, physiological and molecular response and mechanism to overcome the stress condition, we tested the expression of the selected known and novel miRNAs and their predicted target genes in eight different rice varieties with varied degree of drought tolerance and duration. The duration of the drought to achieve a similar level of drought stress varied from 5 (IR64, and TP) to 13 (RS) days (Table 1) when the soil moisture content was in the range of 7–10% from the initial 30–36% in the drought stress treatment pots. Canopy temperature/turgor pressure/stomatal conductance measurements can be measured by thermal imaging (as surrogate). In our experiments, Infrared thermal imaging temperature (IRT) under well-watered control conditions was the least for SD (27.49 ± 0.39 °C), while it ranged from 31–39 °C in other varieties (Fig. 8 A-H). Nearly, 9.5–13.78% increase in IRT was observed after drought stress treatment in all varieties. The cv. RS took maximum number of days (13) to show drought stress symptoms, still, the increase in IRT was only 9.66% (the least increase), indicating its higher drought resilience. Though this quantum of increase was on par with DD (9.76%), the drought stress symptoms could be seen in the latter by 8^th^ day itself. N22, VD and IR64 reached the higher range of 12–13% increase in IRT, but the number of days to achieve the same was 8 days for N22 and VD, while it was 5 days for IR64. In the rice genotypes selected for miRNA validation, we also included TP and KT for which we had earlier standardized the genetic transformation protocols (data not shown) so as to select the appropriate genetic background for overexpression or knockout studies of putative drought responsive/tolerant genes. Our results showed that the response is similar in panels A = B = F, panels C = D and panel G = H (Fig. 8 A-H).

**Table 1:** Thermal imaging under well-watered and drought stress conditions at booting stage in eight rice genotypes with differential response to drought stress

Genotype	IR Temperature			Number of days to Thermal imaging
	**IR Temperature before stress**	**IR Temperature after stress**	**% increase in IR-Temperature after stress**	
Rasi (RS)	31.57 ± 0.12	34.62 ± 0.11	9.66	13
Vandana (VD)	34.46 ± 0.075	39.21 ± 0.54	13.78	8
Nagina 22 (N22)	34.97 ± 0.52	39.34 ± 0.16	12.49	8
DRR Dhan 50 (DD)	31.43 ± 0.59	34.5 ± 0.74	9.77	8
Sahbhagi Dhan (SD)	27.49 ± 0.39	30.76 ± 0.49	11.89	9
Taipei -309 (TP)	35.75 ± 0.59	39.63 ± 0.61	10.85	5
Kitaake(KT)	32.73 ± 0.30	35.95 ± 0.60	9.83	7
IR 64	32.80 ± 0.28	37.00 ± 0.57	12.8	5

**Fig. 9:**
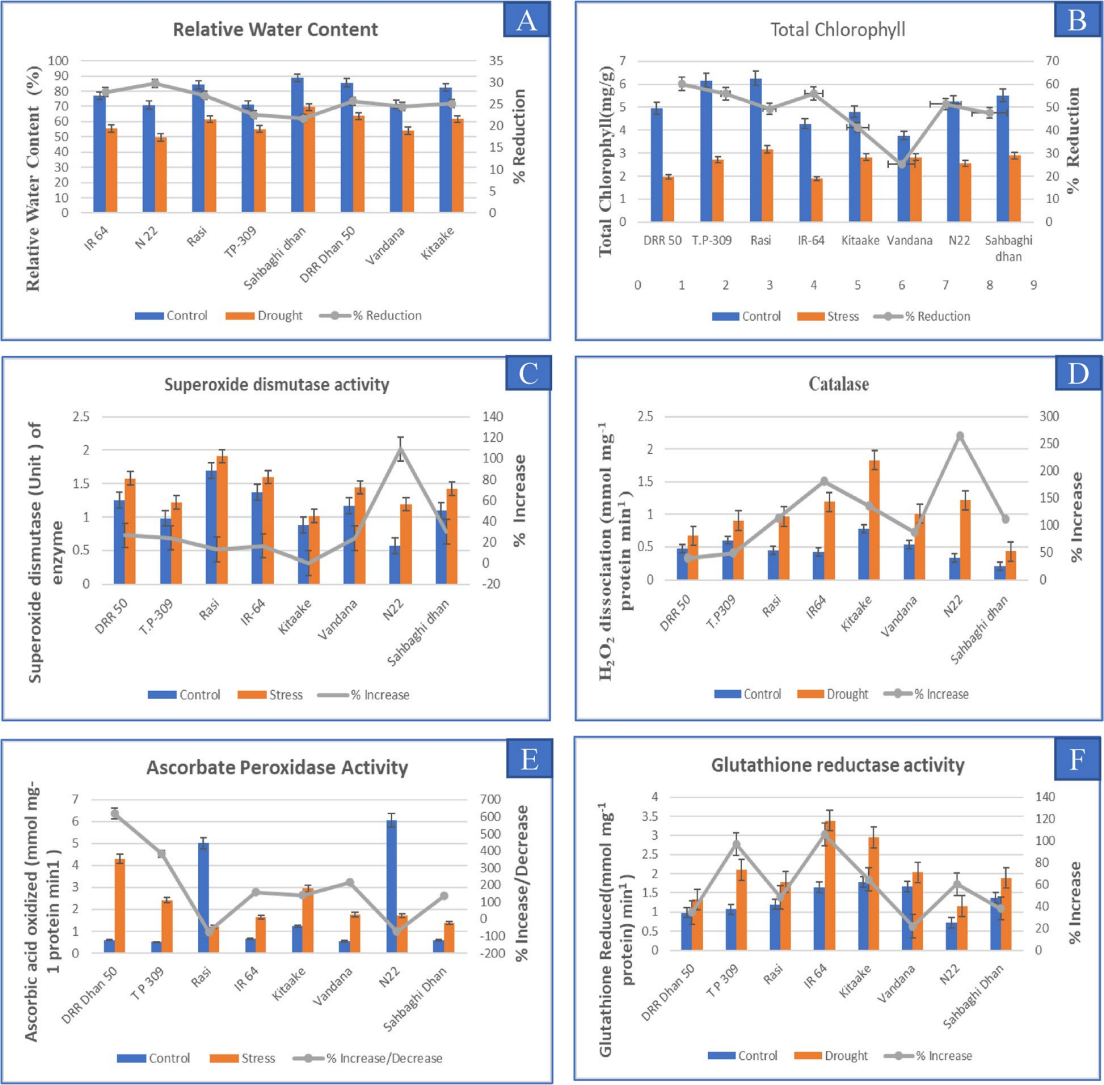
Measurement of various physiological and biochemical parameters under well-watered and drought stress conditions in eight different rice genotypes **A** Relative water content **B** Total chlorophyll content **C**-**F** ROS scavenging enzymes, superoxide dismutase, catalase, ascorbate peroxidase and glutathione reductase activity

Initial RWC across the genotypes was in the range of 70.85–88.74% which reduced to 49–69% after drought stress. Consistently, SD had the highest RWC both before and after stress, while N22 had the least values (Fig. 9A). The percentage reduction in RWC among all genotypes was similar, ranging from 21–29%, suggesting that the drought stress imposed was similar in the entire study material (Fig. 9A). Total chlorophyll content measured showed a significant decrease in all the genotypes tested under drought stress. Highest percent decrease in the total chlorophyll was observed in the DD (60.12%), followed by, IR 64 (55.92%), and TP (55.83%), while the rest of the genotypes showed moderate reduction. The cv. VD showed the least reduction in chlorophyll content (25.16%) (Fig. 9B). Superoxide dismutase activity under drought stress increased in all the genotypes, and the maximum per cent increase was recorded in N22 (109%), followed by SD and DD (Fig. 9C). The least increase in the accumulation of SOD enzyme was seen in TP, IR64, KT, RS and VD. Catalase activity was higher upon exposure to drought stress in all genotypes (Fig. 9D). The highest per cent increase in the concentration of catalase was found in N22 (265%) followed by IR64, KT, SD, RS, VD, TP, and DD (Fig. 9D). Ascorbic acid peroxidase activity was found to increase under drought stress, except in N22, and RS (Fig. 9E). DD showed the highest activity (4.30 mmol mg^−1^ protein min^−1^) followed by TP, VD, IR64, KT, and SD (1.39 mmol mg^−1^ protein min^−1^). Glutathione reductase activity under drought stress was found to be the highest in IR64 (3.38 mmol mg^−1^ protein min^−1^), and the per cent increase in concentration was also the highest in IR64, followed by, TP, KT, N22, RS, SD, and DD with the least accumulation in VD (22.01%) (Fig. 9F). From the assessment of physiological and biochemical parameters, we inferred that RS, VD, N22, DD, and SD showed better drought stress resilience; KT showed moderate resilience; TP was completely sensitive, while IR64 was moderately sensitive to drought stress.

### Expression profiling of known miRNA and their respective target genes

**Fig. 10:**
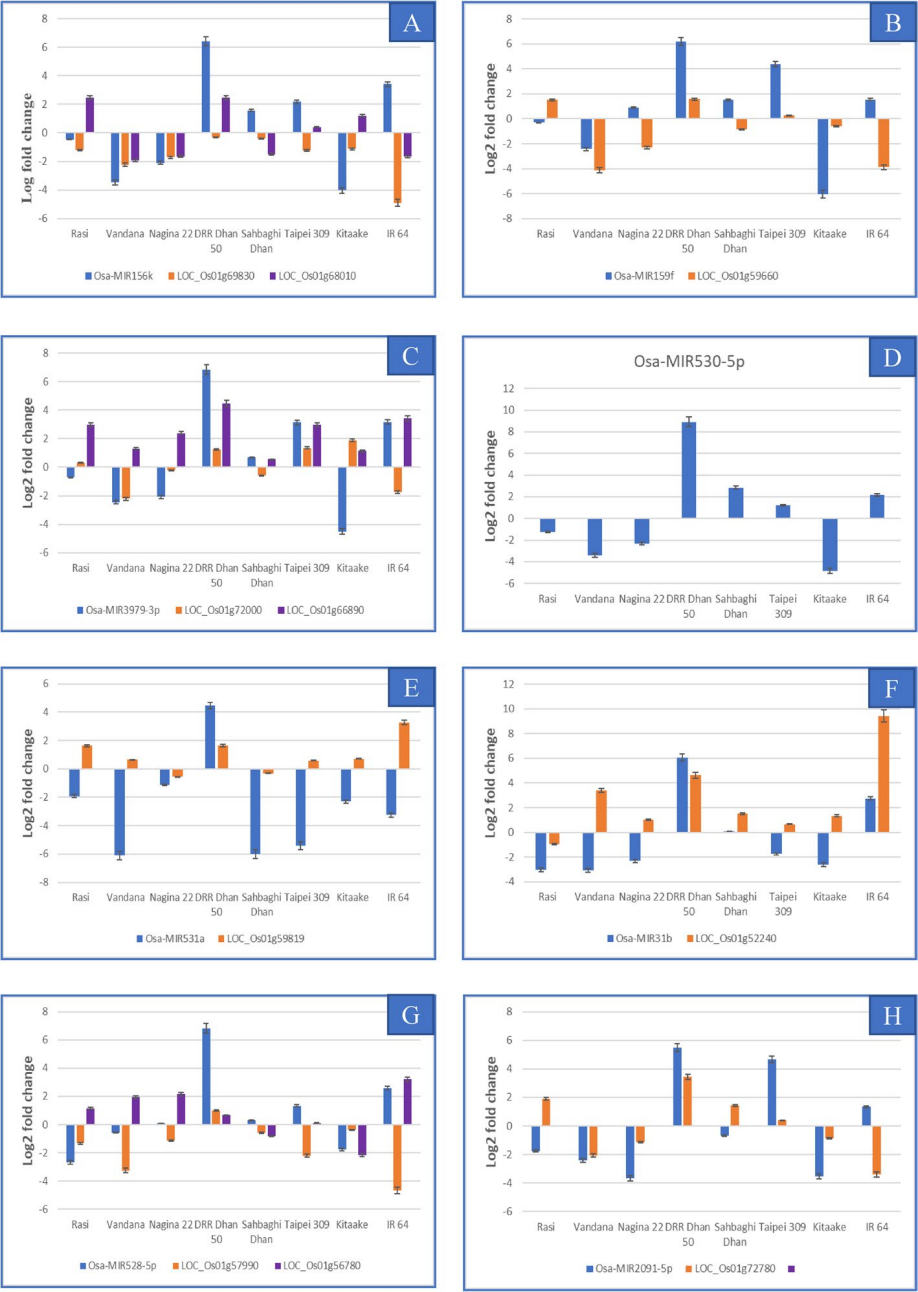
Results of expression profiling of known miRNAs and their target genes **A** Osa-MIR156k and its target genes LOC_Os01g69830 and LOC_Os01g68010; **B** Osa-MIR159f and its target gene LOC_Os01g59660; **C** Osa-MIR3979 and its target gene LOC_Os01g66890 and LOC_Os01g72000; **D** Osa-MIR530-5p; **E** Osa-MIR531a and its target gene LOC_Os01g59819; **F** Osa-MIR531b and its target gene Loc_Os01g52240; **G** Osa-MIR528-5p and its target gene LOC_Os01g57990 and LOC_Os01g56780; **H **Osa-MIR2091-5p and its target gene LOC_Os01g72780

For the identification of drought responsive known miRNAs and target genes, we performed the expression profiling of selected miRNAs-mRNAs in 8 different genotypes at the booting stage. Though each miRNA selected for validation of expression under drought stress had many target genes, we choose only those target genes which were co-localized with the drought stress QTL regions (Fig. 4). Overall, we tested 9 known miRNAs and 17 target genes by expression analysis. Osa-MIR156k showed differential expression in all genotypes under drought stress (Fig. 10A). Maximum fold change was seen in DD (6.4), followed by KT (-4.03), VD (-3.47) and IR64 (3.41). The target genes for Osa-MIR156k, (LOC_Os01g68010: TBC domain containing protein and LOC_Os0169830: OsPL2-SBP box family member) were differentially expressed in all except RS, and TP for LOC_Os01g68010, and in DD and SD for LOC_Os0169830 (< 0.2 fold change). An inverse correlation between transcript abundance of Osa-MIR156k/LOC_Os01g68010 was observed in RS, SD, KT, and IR 64, while for Osa-MIR156k/LOC_Os0169830, only IR64, and TP showed inverse correlation (Fig. 10A).

Osa-MIR159f showed significant fold change under drought stress in all genotype except cvs. RS, and N22 (Fig. 10B). The highest upregulation was observed in DD (6.185), and TP (4.39) while, KT showed maximum downregulation (-sixfold). The target gene, LOC_Os01g59660 (MYB family transcription factor), showed an inverse correlation with the miRNA in N22, SD and IR 64. The lowest fold change (0.285) for the miRNA coupled with the maximum downregulation of the target gene (-4.11) in TP as well as its negligible expression in KT, supported negative correlation between Osa-MIR159f/ LOC_Os01g59660 (Fig. 10B).

Osa-MIR3979 revealed significant differential expression under drought in all genotypes, except, RS and SD (Fig. 10C). It was upregulated in DD, TP, and IR 64, but downregulated in VD, N22, and KT. The fold change (FC) of LOC_Os01g72000 was in appreciable limits in all genotypes except SD while that of LOC_Os01g66890 was in appreciable limit only in DD, KT, and TP. An inverse correlation between the miRNA and both target genes was observed in RS, VD, N22 and KT (Fig. 10 C). Osa-MIR530-5p revealed significant fold change under drought stress in all eight genotypes. Osa-MIR530-5p was upregulated the most in DD (8.91 FC), while the maximum downregulation was in KT (-4.81 FC). The selected target gene, LOC_Os01g699000 (CBS domain-containing protein), could not be validated for expression under drought (Fig. 10D).

From the Osa-MIR531 family, Osa-MIR531a/LOC_Os01g59819 (Os1bglu2—β-glucosidase) and Osa-MIR531b/ LOC_Os01g52240 (chlorophyll a-b binding protein) modules were examined. Both Osa-MIR531a and Osa-MIR531b showed downregulation in all genotypes except DD (Fig. 10 E and F). The maximum downregulation was observed in the VD (-6.11), SD (-6.01), followed by TP (-5.41) for Osa-MIR531a and VD (-3.1) and RS (-3.02) for Osa-MIR531b. Expression of Osa-MIR531a/ LOC_Os01g59819 showed either perfect inverse correlation (VD, IR 64, RS, TP and KT) or slight leaky expression in SD (Fig. 10E). Inverse correlation between miRNA and the target gene was observed in VD, N22, TP and KT for Osa-MIR531b/ LOC_Os01g52240 (Fig. 10F).

Osa-MIR528-5p was drought stress-responsive only in DD, IR64, RS, and KT (Fig. 10G). LOC_Os01g57990 (expressed protein), one of the target genes of Osa-MIR528-5p, was downregulated in all genotype except DD (0.99 FC). The maximum FC was in IR64 (-4.66), followed by VD (-3.26), while in SD and KT the FC was very low. The other target gene, LOC_Os01g56780 (plus-3 domain containing protein), was upregulated in all genotypes except KT (-2.18) and SD (-0.81). An inverse correlation between miRNA and LOC_Os01g56780 was observed in RS, VD, SD while either the gene or the miRNA showed negligible differential expression in N22, TP, and SD (Fig. 10G).

Osa-MIR2091-5p expression was upregulated in DD, TP, and IR64 while it was downregulated in rest of the five genotypes under drought stress (Fig. 10H). Maximum FC was observed in DD (5.49), followed by TP (4.67) in case of upregulation, and N22 (-3.68) and KT (-3.55), in case of downregulation. LOC_Os01g72780 expression ranged from -3.4 (IR64) to + 3.45-fold (DD). Inverse correlation between MIR-2091-5p/ LOC_Os01g72780 was observed in RS, SD, and IR 64 (Fig. 10H).

**Fig. 11:**
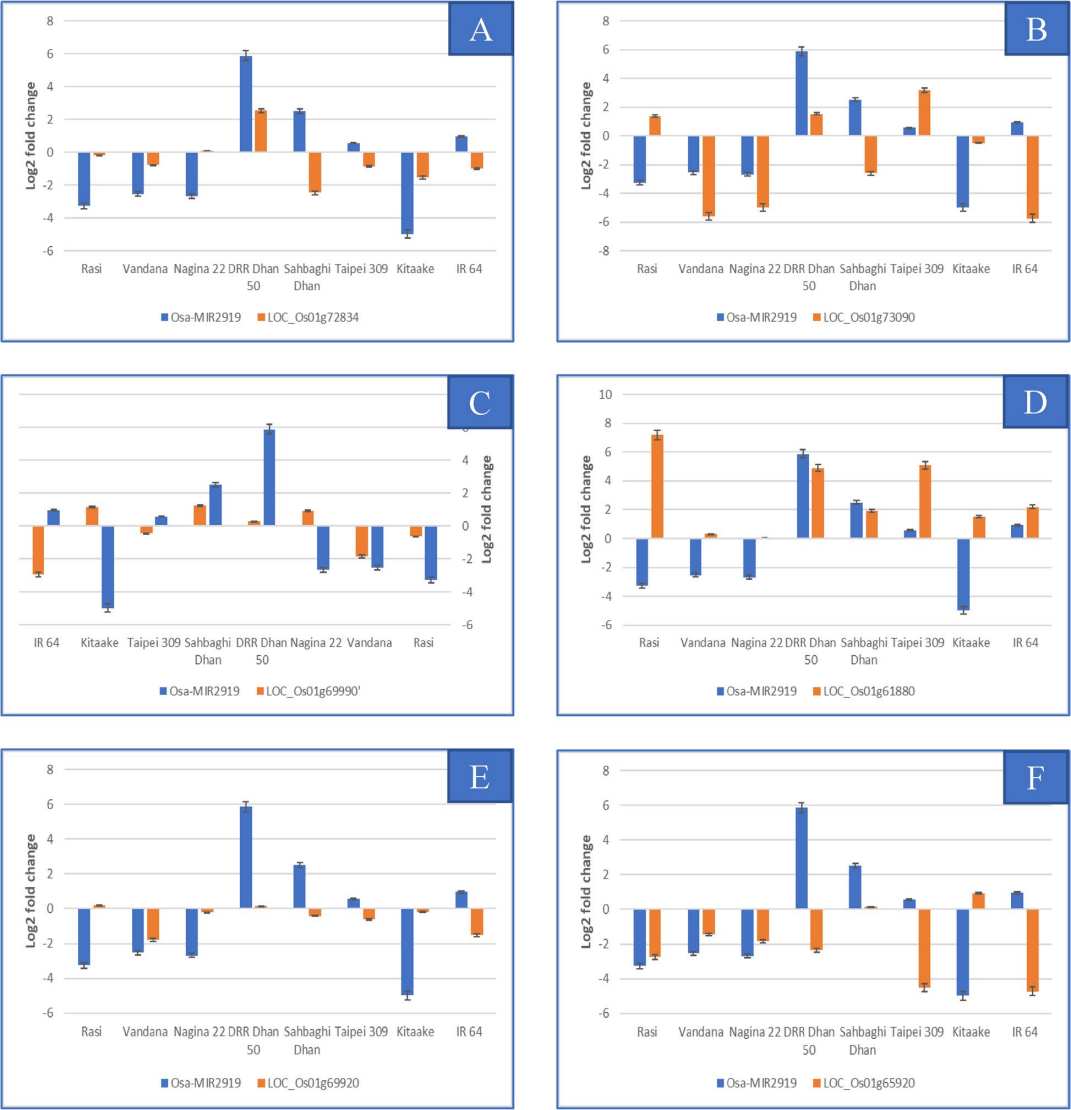
Expression profiling of Osa-MIR2919 and its target gene; **A** Osa-MIR2919 and its target gene LOC_Os01g72834; **B** Osa-MIR2919 and its target gene LOC_Os01g73090; **C** Osa-MIR2919 and its target gene LOC_Os01g69990; **D** Osa-MIR2919 and its target gene LOC_Os01g61880; **E** Osa-MIR2919 and its target gene LOC_Os01g69920; **F **Osa-MIR2919 and its target gene LOC_Os01g65920

Differential expression of Osa-MIR2919 was observed in all the genotypes under drought stress. It was upregulated in DD (5.88-fold) followed by SD, IR 64 and TP, while it was downregulated the maximum in KT (-4.98) followed by RS, N22 and VD (Fig. 11 A-F). Osa-MIR2919 had the maximum number of target genes (6) in the major drought QTLs which included LOC_Os01g72834, LOC_Os01g73090, LOC_Os01g69990, LOC_Os01g61880, LOC_Os01g69920 and LOC_Os01g65920 encoding for RNA recognition motif containing protein, expressed protein, GYF domain containing protein, respiratory burst oxidase, histidine kinase and F-box/LRR-repeat protein 2, respectively. Of these, the cv. RS showed consistent inverse correlation for four of the six target genes LOC_Os01g73090, LOC_Os01g61880, LOC_Os01g69990 and LOC_Os01g69920 and negligible differential expression for LOC_Os01g72834 (Fig. 11). IR64 also showed either negligible differential expression of the target gene (LOC_Os01g69990), or inverse correlation of the target genes (LOC_Os01g73090, LOC_Os01g69990, LOC_Os01g61880, and LOC_Os01g69920), with Osa-MIR2919, under drought. KT showed inverse correlation for three target genes LOC_Os01g61880, LOC_Os01g69990 and LOC_Os01g65920 followed by N22 which showed the inverse correlation between the expression profiles of Osa-MIR2919 and three target genes, namely, LOC_Os01g72834, LOC_Os01g61880, and LOC_Os01g69990 (Fig. 11).

**Fig. 12:**
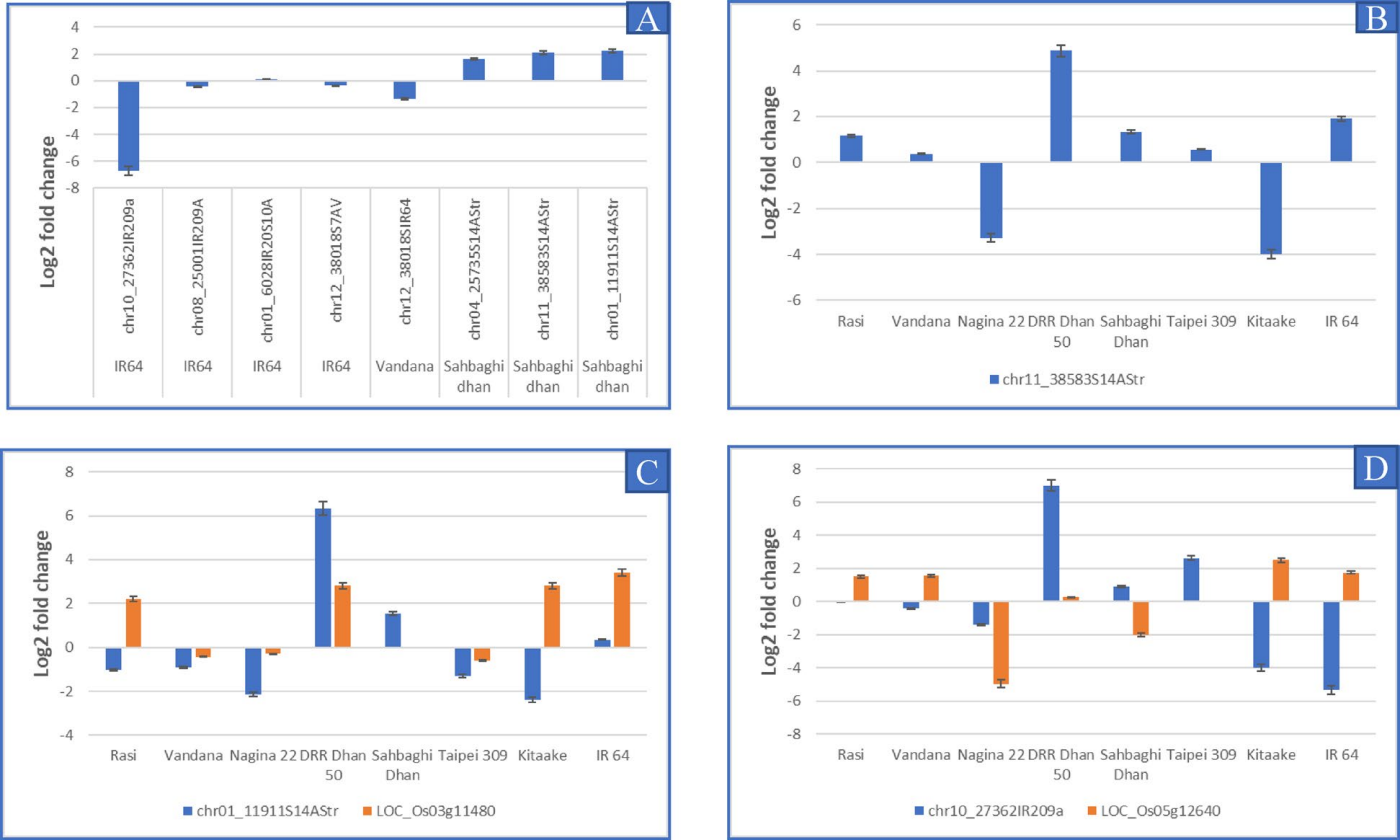
Expression analysis of novel miRNAs and target genes; **A** Expression analysis of seven novel miRNAs in their respective genotypes; **B** chr11_38583S14AStr in eight genotypes; **C** chr01_11911S14AStr and its target gene LOC_Os03g11480; **D** chr10_27362IR209a and its target gene LOC_Os05g12640

DD behaved differently from rest of the genotypes in that it showed the highest upregulation for nine known and three novel miRNAs tested under drought stress in the present study (Figs. 10, 11 and 12). Of the 17 target genes analysed for the nine known miRNAs, an inverse correlation was observed only for miR156k/LOC_Os0169830 and miR2919/ LOC_Os01g65920 while negligible differential expression of the target gene was found for the both the target genes of miR528.

### Expression analysis of the novel miRNAs identified and their predicted target genes

The nine novel miRNAs selected for validation were first checked for their presence in the genotype from which they were detected. Only seven out of the nine novel miRNAs could be amplified in the control sample while the remaining two (chr01_4341IR20S10A, and chr02_20631IR20S9B) could not be amplified (Supplementary Fig. 4A). Of these, only 5 were found to be significantly expressed under drought (Fig. 12A). chr10_27362IR209A showed maximum downregulation in IR64 (-6.69-fold), chr12_38018S7AV in VD (-1.35), while chr04_25735S14AStr (1.64), chr11_38583S14AStr (2.10) and chr01_11911S14AStr (2.25) were upregulated in SD (Fig. 12 A). On the basis of drought responsiveness of the novel miRNAs, we selected chr01_11911S14AStr and chr11_38583S14AStr from SD and chr10_27362IR209A from IR 20 for expression analysis in all the selected genotypes.

Expression of chr11_38583S14AStr was upregulated in all genotypes except N22 (-3.28 fold change) and KT (-4.0125 FC) (Fig. 12B). However, the target gene chosen for this miRNA (LOC_Os01g68790) could not be amplified. chr01_11911S14AStr was differentially expressed in all genotypes, except, IR64 (Fig. 12C). It was upregulated in DD (6.33), and SD (1.53). In SD, expression of the target gene LOC_Os03g11480 (expressed protein) was not detected, while in the other genotypes it showed either upregulation or negligible downregulation. An inverse correlation between the miRNA and the target gene was found in RS and KT while it was completely absent in DD. In the remaining genotypes, either the differential expression in the gene or the miRNA was negligible (Fig. 12C). Expression of chr10_27362IR209A miRNA was upregulated in DD (6.98), and TP (2.62), whereas IR64 showed the highest decrease in the expression (-5.353 fold), followed by, KT and N22 (Fig. 12D). An inverse correlation could be observed between the miRNA and the target gene (chr10_27362IR209A /LOC_Os05g12640- BURP domain containing protein) in VD, SD, KT and IR 64 while others showed negligible differential expression in either the gene or the miRNA.

### Validation of drought responsive miRNAs in environmental information processing

Based on the target gene analysis, the major miRNAs involved in recognizing environmental cues were Osa-MIR2919, Osa-MIR156k and chr12_39994IR20S10A. LOC_Os01g65920, involved in the FoxO signaling pathway and a target of Osa-MIR2919 was found to be two fold and four fold downregulated in N22 and IR64 under drought stress (Fig. 11F). The targets of Osa-MIR156k and Osa-MIR2919, LOC_Os01g21810 and LOC_Os01g69920, respectively, encode for histidine kinase and is thus involved in the perception of cytokinin which ultimately results in cell division and shoot initiation. Of these two genes, LOC_Os01g69920 is a part of the drought QTL and it was found to be downregulated in all the eight genotypes at booting stage (Fig. 11E). Osa-MIR2919 also targets LOC_Os02g14130, involved in the brassinosteroid biosynthesis, and in RiceMetaSys this gene was found to be upregulated in N22 but downregulated in IR64.

## Discussion

miRNAs work on secondary level by regulating the genes involved in phenological, physiological and molecular changes and a number of investigations on miRNA-omics modulating the plant responses to drought, cold, salinity, high temperature, plant type and architecture have been reported in rice [42–46]. In the present study, the differentially expressed miRNAs under drought stress at booting stage were mined from small RNA sequencing data, their target genes were predicted using multiple bioinformatics tools, and further verified by experimental evidences from degradome datasets and RiceMetaSys. Finally, the broad regions pertaining to major QTLs that govern yield under drought stress were examined to identify relevant miRNA-mRNA modules, if any, by combining expression analysis, allele mining and protein structural changes. All the results were integrated to identify the most promising miRNAs and their target genes for enhanced drought stress tolerance.

Leaf/canopy temperature of the plant can be used as the indicator of stomatal aperture which indicates water responses of the plant toward the stress as leaf temperature is directly related to the stomatal conductance [47]. The genotypes which could resist the increase in temperature or take longer duration to increase the temperature could be considered as drought tolerant while genotypes with rapid increase in temperature under drought could be considered as drought sensitive. Our results indicated that SD, RS, VD, DD, N22 and KT were drought tolerant whereas IR64 and TP were drought sensitive. Our results provided evidence for the different mechanisms of drought tolerance operating in SD (higher RWC under drought), RS (better canopy temperature adjustment), N22 (better ROS mechanism) and VD (a combination of multiple mechanisms). The better ROS mechanism of N22 has been reported earlier [39].

SD is a well-known drought tolerant donor which has been the least explored in terms of molecular basis of drought stress tolerance. Our study revealed that the miRNA modulation of SD is unique compared to the other two genotypes (Fig. 1B). Among the known miRNAs, all members of the Osa-MIR156 family were found to be downregulated under drought in IR20 and VD, but upregulated in SD and this pattern was also validated by expression profiling. Osa-MIR156j was has been reported to be highly abundant in N22 [34] but downregulated in IRAT109 under drought [33]. Differential expression of Osa-MIR821 was exclusive to the drought sensitive cv. IR 20 in which all members of this family were found to be downregulated in the present study. Banerjee et al., [48] reported that Osa-MIR821 had a role in controlling the level of amino acid, valine. Similarly, Osa-MIR528 was found to be downregulated in IR20 but upregulated in SD and VD under drought. Balyan et al., [41] reported downregulation of Osa-MIR528 in drought sensitive genotypes IR 64 and Pusa Basmati 1 and upregulation in tolerant genotypes N22 and VD. Constitutive expression of this Osa-MIR528 in bentgrass lead to alteration in development of plant and enhanced tolerance to salinity and nitrogen starvation [49].

The target genes of Osa-MIR156k belonged to the OsSPL family. The OsSPL members were reported to be involved in the regulation of meiotic fate acquisition in rice [50] and male fertility under high temperature by modulation of the flavonoid pathway [51]. Degradome results for the target genes of this miRNAs was also supported by RiceMetaSys: out of the six target genes, two, LOC_Os01g69830 (OsSPL2), and LOC_Os07g32170 (OsSPL13), were differentially expressed under drought conditions in at least one dataset while the former was validated by expression analysis by us (Fig. 10A). Though Osa-MIR159f had multiple targets, only two genes, MYB transcription factor (LOC_Os01g59660) and DUF630/DUF632 domains containing protein, were found in multiple degradome libraries and RiceMetaSys. MYB transcription factor (LOC_Os01g59660) has a well-known role in plant development and responses to abiotic stress which is brought about by modulating the biosynthesis of secondary metabolites [52]. DUF630/DUF632 domain has an important role in controlling the leaf rolling of plants through *REL2* [53] and stomatal patterning through *RSD1* gene in rice [54]. Osa-MIR528 targets both LOC_Os06g37150 (L-ascorbate oxidase precursor) and LOC_Os06g06050 (OsFBL27—F-box domain and LRR containing protein) from the QTLs regions. Of these, only L-ascorbate oxidase precursor had support from RiceMetaSys. The role of L-ascorbate in mitigating excessive cellular reactive oxygen species activities induced by several abiotic stresses is well documented [55]. OsFBL27—F-box domain and LRR containing protein is involved in panicle and seed development and regulated by light and abiotic stress in rice [56] and hence can be one of the most probable target genes for this miRNA during booting stage under drought stress.

For the novel miRNA chr01_6028IR20S10A, 10 genes were available within the QTL, but only two, LOC_Os01g66970 (zinc finger, C3HC4 type domain containing protein) and LOC_Os01g61010 (nodulin) were supported by RiceMetaSys. Zinc finger C3HC4 type domain containing protein has been found to modulate the growth and development, signalling networks and in various abiotic stresses in Arabidopsis etc. [57]. Nodulin are involved in regulating membrane transporters, water permeability under osmotic stress, and nodulin modulated phosphorylation controls the cellular transport during osmotic adaptation [58, 59]. Out of the eight target genes for chr04_25735S14AStr, three were supported by RiceMetaSys, LOC_Os01g59160, LOC_Os01g62480 and LOC_Os12g25170. LOC_Os01g59160 which encodes Ubiquitin-Associated (UBA) domain protein has been found to be associated with controlling the heading date and grain weight in rice [59]. LOC_Os01g62480 encodes laccase2 and heterologous expression of *Populus euphratica* laccase (LAC2) gene enhanced drought tolerance in Arabidopsis and *Populus* by modulating the xylem structure, thickening of secondary cell wall, increasing the fibre cell length and stem tensile strength which ultimately improve water transport capacity of plants [60].

**Fig. 13:**
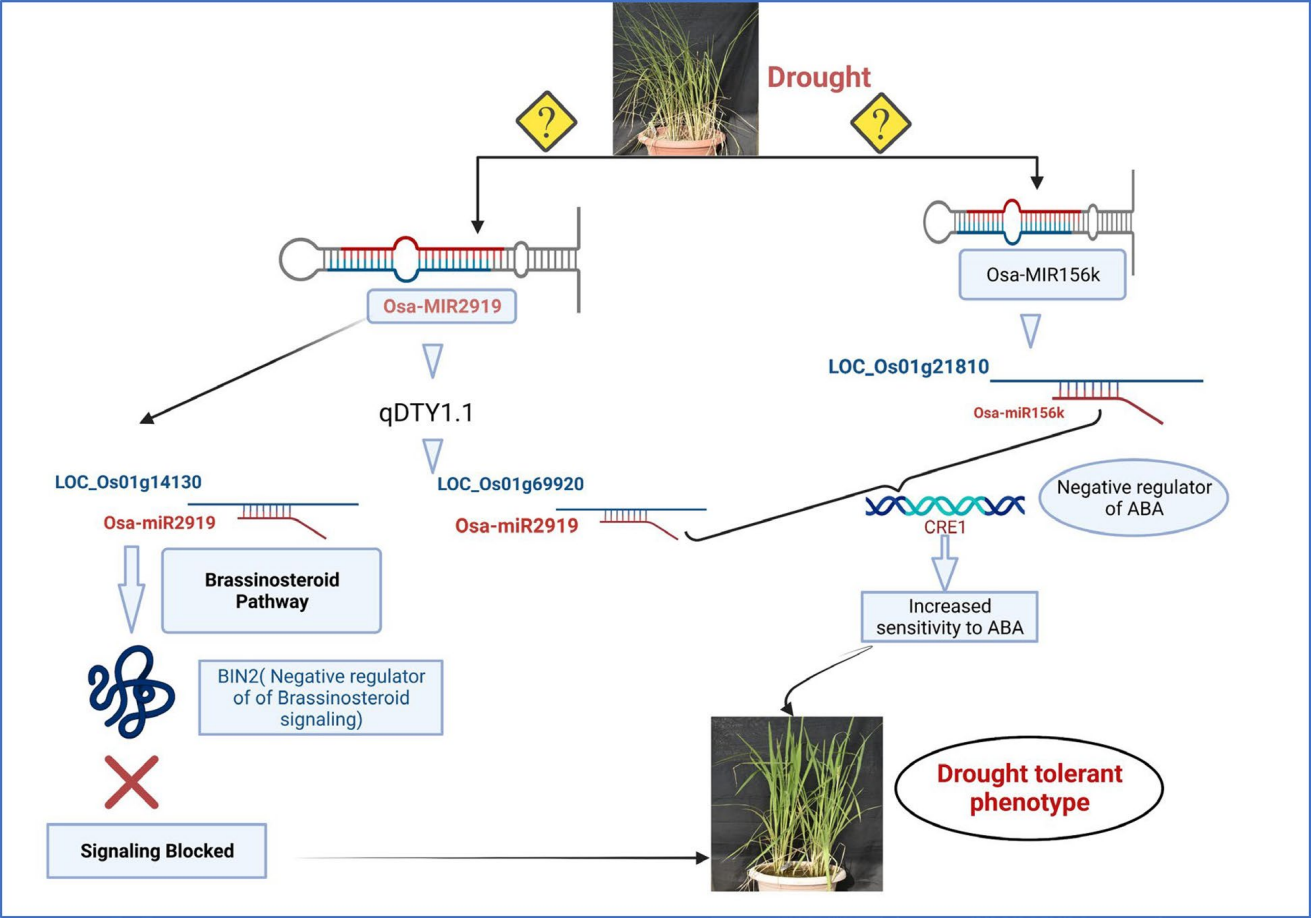
Proposed model of drought tolerance at booting stage by Osa-MIR2919 and Osa-156 k (Using BioRENDER)

Among the known miRNAs, 11 miRNAs had target genes co-located within the drought QTL regions which included Osa-MIR156k, Osa-MIR2091-5p, Osa-MIR2919, Osa-MIR3979-3p, Osa-MIR528-5p, Osa-MIR530-5p, Osa-MIR531a, Osa-MIR531b, Osa-MIR820a, Osa-MIR399j, and Osa-MIR396a-3p. Of these, Osa-MIR2919 had the maximum number of target genes (19) within the QTL of which LOC_Os01g15770 (transmembrane protein 136), LOC_Os04g33990 (hairpin-induced protein 1 domain containing protein) and LOC_Os10g14920 (integral membrane protein DUF6 containing protein, expressed) were detected in multiple degradome libraries. Target genes co-located within the QTL and supported by RiceMetaSys for this miRNA were LOC_Os01g61880, LOC_Os01g69920, LOC_Os01g65920, LOC_Os01g63620, LOC_Os01g65410, LOC_Os01g67490, and LOC_Os01g72000. The pathway analysis identified LOC_Os01g65410 (serine hydroxy methyl transferase), involved in various metabolic activity especially secondary metabolites and amino acids, LOC_Os01g65920 (F-box/LRR-repeat protein 2) involved in degradation, FoxO signaling pathway, mTOR signaling pathway and cell cycle, and LOC_Os01g69920 (histidine kinase) involved in plant hormone signal transduction as the major candidates. Specifically, in the cytokinin biosynthesis pathway, it targets the receptor of cytokinin, CRE1, and has a role in cell division and shoot initiation. Cytokinin signalling genes expression usually lowered in response to drought [61, 62]. Lowered cytokinin content and signaling lead to increased sensitivity to ABA-establishing CK as antagonist of ABA [63] and reduced shoot growth [64]. In our study, under drought stress, LOC_Os01g69920 was found to be highly downregulated in IR64 and TP309 but less so in rest of the genotypes including N22 and KK. LOC_Os10g21810 which also encodes histidine kinase, but regulated by Osa-MIR156k, was found to be upregulated under drought in IR64. Another target of Osa-MIR2919, LOC_Os02g14130 that play a role in brassinosteroid biosynthesis pathway, encodes BIN2 which is negative regulator of brassinosteroid signalling. We got experimental evidence from RiceMetaSys for this gene wherein it was upregulated in drought tolerant N22 but downregulated in drought sensitive IR64. Thus, evidences for modulation of both cytokinin and brassinosteroid signalling by respective miRNA-mRNA modules under drought stress emerged from our study (Fig. 13). It is pertinent to note here that for Osa-MIR2919, per se, no experimental evidence under drought stress is available so far. Possible involvement of MIR2919 in controlling flavonoid biosynthesis pathway in turmeric [65] and submergence tolerance in rice [66] have been reported. This is the first report on this miRNA-mRNA module under drought stress.

## Conclusion

The present study led to the identification of 18, 23, and 12 known and 20, 14, and 6 novels differentially expressed miRNA in IR20, SD and VD respectively under drought at booting stage. Degradome datasets were found to be the more reliable tools for target gene prediction of miRNAs. Through expression analysis of the selected drought stress responsive miRNAs, we could validate nine known and three novel miRNAs, Of these Osa-MIR3979, Osa-MIR2919, Osa-MIR531a and Osa-MIR531b are being reported for the first time for drought stress in rice. We narrowed down 61 target genes in the three major drought QTLs regions that are modulated by the drought responsive miRNAs. Of these, 20 were found to be the most promising candidate target genes as supported by the degradome datasets. The study could establish miRNA-mRNA module for Osa-MIR159f/LOC_Os01g59660, Osa-MIR3979-3p/ LOC_Os01g72000/LOC_Os01g66890, Osa-MIR531a/ LOC_Os01g59819/, Osa-MIR531b/LOC_Os01g52240, Osa-MIR528-5p/ LOC_Os01g57990/LOC_Os01g56780 and, Osa-MIR2919/LOC_Os01g72834/ LOC_Os01g61880. The present study established the contribution of genes from the three major drought tolerant QTLs with their miRNA regulators. Of these, the role of Osa-MIR2919 through 19 target genes especially through cytokinin and brassinosteroid modulation is being reported for the first time. They were found to be supported by multiple evidences such as differential expression in degradome libraries, drought specific transcriptome datasets and structural and expression differences in the relevant rice germplasm.

## Methods

### Plant materials, growth conditions and drought stress treatment

In the present study, nine rice varieties, namely, Nagina 22 (N22), IR 64 (IR64), Rasi (RS), DRR Dhan 50(DD), Vandana (VD), Sahbhagi Dhan (SD), Kitaake (KT), Taipei 309 (TP), and IR 20 (IR20), were used. For small RNA sequencing, three genotypes, namely, VD, SD and IR20 were used. All the nine genotypes, except IR20, were used in the miRNA and their target gene expression study. Seeds of the selected rice genotypes were surface sterilized and soaked in water overnight. The imbibed seeds were kept on a water-soaked paper towel to induce seed germination; after germination, the seeds were sown on a nursery bed prepared in the field. After 21 days of sowing, the young seedlings were transferred to 15-inch diameter pots, which were kept in the ‘abiotic stress phenotyping facility’ of the National Institute for Plant Biotechnology, New Delhi. To impose drought stress on the emerging panicles, the water supply was stopped at the initiation of booting. This was identified based on the swelling of the base of the leaf sheath and the extension of the flag leaf.

For small RNA-seq studies, flag leaf samples were collected after 8 days of drought stress, when the relative water content (RWC) reached 60.05%, 72.6%, and 70.62%, down from 80.03%, 89.57%, and 90.4%, respectively, in IR20, VD, and SD, indicating a 19–25% reduction in RWC. The eight genotypes used in the miRNA and their target gene validation studies, showed variation in their days to booting. Hence, the drought stress was initiated at different time point for different genotypes, according to their days to booting. The sampling of flag leaves from the drought stress treatment was done based on the soil moisture content of the individual pots. The soil moisture content in pots at sampling ranged from 7–10% compared to the initial 30–36% (as measured by gravimetric method). The samples were collected and immediately frozen in liquid nitrogen and stored at -80 °C for future experiments. Simultaneously, a set of plants with regular and adequate watering was maintained as control. There were three independent replications for control and drought stress treatments. Three pots per replication were maintained and each pot had two plants to facilitate the assessment of drought stress response and small RNA sequencing.

### Physiological and biochemical parameter measurements

Canopy temperature, relative water content (RWC) and ROS scavenging enzyme activity were measured under both drought stress and control treatments. To measure the canopy temperature, infrared thermal images were taken with the help of Infrared Camera (Fluke TiX620), with a resolution of 640 × 480 pixels. The camera measures the temperature in the range between -40 to 120 °C. The images obtained were processed with the help of SmartView Classic 4.4 software. RWC of rice leaves was calculated using the formula proposed by Barrs and Weatherly., [67] and described by Lima et al., [68], RWC = {(fresh weight—dry weight) / (turgid weight—dry weight)} X 100. Total chlorophyll was measured by DMSO method using Arnon’s equation, and accordingly, chlorophyll content was calculated [69]. In addition to this, several antioxidant enzymes, namely superoxide dismutase (SOD; EC 1.15.1.1), glutathione reductase (GR; EC 1.6.4.2), ascorbate peroxidase (APX; EC 1.11.1.1), and catalase (EC 1.11.7.6) were measured.

For all the measurements other than the canopy temperature, flag leaf samples collected in triplicate were used. For enzyme assays, leaf samples collected and frozen in liquid nitrogen were used. Only the RWC was measured immediately after sample collection. Data analysis was done according to one way ANOVA (*p*-value threshold < 0.05) and the mean comparisons were made using Tukey’s test.

### Small RNA library construction and sequencing

Total RNA from the flag leaves of IR20 (known drought stress sensitive genotype), VD and SD (known drought stress tolerant genotypes) was isolated. Spectrophotometry was used to quantify and check the purity of the RNA using a Nanodrop ND8000 (Thermo Scientific, USA) and a 1.5% denaturing agarose gel. The sRNA library was prepared according to the manufacturer’s protocol (TruSeq RNA Library Prep Kit v2) and single end Illumina sequencing was performed. The sampling for sequencing was done in two replicates, so a total of 12 libraries (3 genotypes* 2 treatments* 2 replications) were generated. Raw reads of sRNA sequencing are available in SRA database with Project id PRJNA851230.

### Small RNA analysis and identification of miRNAs

Sequencing data from the 12 libraries was processed using a comprehensive analysis pipeline. The quality of raw reads was checked using fastqc [70], following which adapter trimming was performed by using the Trim_galore package [71] with the following settings: 1) All reads with a quality score of less than 20 were eliminated. 2) Reads with read length less than 17 bps and more than 30 bps were discarded. The filtered reads were mapped on the *Oryza sativa* genome (MSU version 7.0) using the SHRiMP package [72] with default mapping parameters. For the identification of known and novel microRNAs, miRDeep2 version 0.1.3 Friedlander et al., [73] was used. The minimum free energy and prediction of secondary structure of novel miRNAs were done using the RNAFold tool from ViennaRNA package [74]. For identification of differentially expressed novel and known miRNAs, the Deseq2 package in R was used, which takes the raw read count of miRDeep2 as input. The basic criteria considered for a sRNA as a miRNA, given by [75–78], were used to identify the miRNAs. In total, the following eight criteria were applied to identify the precursor: 1) A minimum of 3 read counts must be present for the sRNA, 2) The sRNA has been mapped to the precursor with an exact sequence match, 3) The precursor length must be at least 60 nt long, 4) The precursor forms a hairpin structure when subjected to folding using the RNAFold software from the Vienna package 5) Only one of the hairpin's arms is occupied by the sRNA sequence 6) The sRNA sequence on the opposing strand has no more than six mismatches 7) The A + U concentration ranges from 30 to 70%, and 8) The minimal free energy (MFE) is less than or equal to -15 kcal/mol.

### Target prediction

Target prediction of the selected known and novel miRNAs was done by using two different tools, namely, the psRNATarget web-based server [79] and psRobot [80], against *Oryza sativa* transcripts from MSU Rice genome annotation, version 7, with the default parameters. Only those targets with expectance or score of ≤ 3 were considered for further analysis. We further used the nine publicly available rice degradome datasets, namely SRR032098, SRR032097, SRR521269, SRR034102, SRR039716-20, SRR7706267, SRR7706268, SRR7706269 and SRR7706270 to narrow down the drought stress specific targets. For this, the raw reads were downloaded from the SRA database and processed to remove the adapter sequence and low-quality reads. The remaining high-quality reads were used for target prediction using the Cleaveland pipeline [81]. Output of the Cleaveland pipeline is described in five categories, 0 to 4, of which we considered only those targets falling under the categories 0, 1, and 2. To confirm the nature of the drought responsiveness of the predicted miRNA target genes, we made use of the publically available database, RiceMetaSys, which is a comprehensive inventory of drought stress responsive genes (http://14.139.229.201/RiceMetaSys/; [36]. Finally, we compared the genes present in the three major QTLs regions for yield under drought stress, namely, qDTY1.1, qDTY 3.1 and qDTY 12.1 [22, 23, 26]. The genes in the QTLs intervals were retrieved from the gramene database (https://www.gramene.org/). The most promising miRNA target genes that co-localized in the QTLs regions were chosen for validation by qRT-PCR based expression analysis.

### GO enrichment and pathway analysis

GO enrichment of the genes obtained by RiceMetaSys was done using Blast2go [82] and pathway analysis was carried out by the Blastkola (https://www.kegg.jp/blastkoala/; [83–85]. For Venn diagrams, Venny (https://bioinformatics.psb.ugent.be/webtools/Venn/) was used. The entire workflow from the analysis of the small RNA-seq data to the identification of miRNAs and their target genes followed by their validation is summarised in Supplementary Fig. 1.

### Sequence variation, promoter analysis and protein modelling

To find whether there are any allelic differences in Osa-MIR2919 and its target genes between a pair of drought stress tolerant and sensitive genotypes, a well-known drought tolerant donor N22 and a popular cultivar IR64 with sensitive response at field level were used. Multiple alignment tool Clustal Omega was used for SNP identification from the sequence information on these two genotypes available with us [86, 87]. For cis element analysis in promoter region 2 kb upstream sequence were retrieved and analysed in PlantCARE [88]. Secondary and tertiary protein structure predications were made using I-TASSER and Alphafold, respectively [89, 90].

### Estimation of abundance of miRNA by qPCR

Total RNA from the flag leaves was isolated by the IHBT protocol [91]. To get rid of the genomic DNA contamination, DNase (Fermentas, USA) treatment was performed. Expression profiling of the selected miRNAs was done using the miR-X qPCR kit (Clonetech, USA). Total RNA was polyadenylated and reverse transcribed using Poly(A) polymerase and SMARTTM M-MLV reverse transcriptase, which were included in the kit's mRQ enzyme mix. To quantify the miRNA level, qPCR was performed in a 10 µl reaction mixture constituted by 5 µl Applied Biosystems™ SYBR Green Master Mix, 1 µl cDNA sample (50 ng), 0.5 µl miRNA specific primer (10 pmol/ µl), 0.5 µl mRQ 3’ primer (10 pmol/ µl), and 3 µl nuclease free water using Aria MX Real-time PCR system (Agilent Technologies, USA). The primers of U6 gene were used as a normalizer. Delta-delta Ct method [92] was used for quantification of the cellular abundance of a miRNA, and the result was expressed as fold change in abundance of the miRNA in the drought treated plants over that of the respective control plants.

### Expression profiling of the target genes

For expression profiling of the predicted and selected target genes, we isolated the total RNA from the flag leaf of all the genotypes by the IHBT protocol [91] and treated it with DNase. Total RNA was converted into cDNA using SuperScript® III First-Strand Synthesis followed by cDNA reverse transcription kit (Thermo Fisher, USA). qRT-PCR was performed using the Applied Biosystems™ SYBR Green Master Mix in the AriaMx Real-time PCR System (Agilent, USA). All qRT-PCR experiments were performed for the identified target genes of the selected miRNAs, in all the eight rice genotypes, with three biological and three technical replicates. The final reaction volume of 10 μl consisted of 50 ng cDNA, 0.5 μM of forward and reverse primer each, and 5 μl of Applied Biosystems™ SYBR Green Master Mix reagent. Actin gene was used as a normalizer or internal control. The relative expression of the genes was calculated based on the formula (Ratio = 2^−ΔΔCt^) under control and drought conditions [92].

### Supplementary Information

**Additional file 1.** [40, 71, 93–99].

**Additional file 2.**

**Additional file 3.**

**Additional file 4.**

**Additional file 5.**

**Additional file 6.**

**Additional file 7.**

## Data Availability

The information for analysis is in the Supplemental Tables. The raw reads of sRNA sequencing are available in SRA database with Project id PRJNA851230.
