# Supplementary material for: Integration of miRNA dynamics and drought tolerant QTLs in rice reveals the role of miR2919 in drought stress response
Source: BMC Genomics. 2023 Sep 6;24:526. doi: 10.1186/s12864-023-09609-6 (PMC10481553; doi:10.1186/s12864-023-09609-6)
Supplement: Supplementary file 1 — Additional file 1. [40, 71, 93–99]. [file 12864_2023_9609_MOESM1_ESM.docx]

| **Genotype** | **Stage** | **Findings** | **Reference** |
| --- | --- | --- | --- |
| *Oryza sativa* cv. *Japonica* | Vegetative stage | Identified two drought induced miRNA in which miR169g was confirmed as the only member induced by drought which was expressed more prominently in roots | Zhao et al., 2007 |
| IRAT109 | Tillering stage, Inflorescence forming  stage (booting) | Identified 30 miRNAs; 11 were down-regulated (miR170, miR172, miR397, miR408, miR529, miR896, miR1030, miR1035, miR1050, miR1088, miR1126) and eight were up-regulated miRNAs (miR395, miR474, miR845, miR851, miR854, miR901, miR903, miR1125) were revealed for the first time to be induced by drought stress in plants | Zhao et al., 2010 |
| Nipponbare | Reproductive stage | Identified 18, 15, and 10 miRNAs that were regulated by drought, cold and salt stress conditions, respectively | Barrera et al., 2012 |
| Vandana, Aday Sel and IR64 | Vegetative stage | Identified four miRNAs, namely, osa-miR397a/b, osa-miR398b, osa-miR408-5p, and osa-miR528-5p which were down-regulated in the drought-tolerant rice varieties (Vandana and Aday Sel) but up-regulated in the drought-sensitive IR64 | Cheah et al., 2015 |
| Nagina 22 | Heading stage  (Flag leaves, spikelets and roots) | Identified 71 novel miRNAs in the drought-tolerant *‘*aus*’* rice cv. Nagina 22. These were validated based on precursor hairpin structure, small RNA mapping pattern, ‘star’ sequence, conservation, and identification of targets based on degradome data. | Mutum et al., 2016 |
| IR64 and its drought tolerant BIL (IR77298-14-1-2-10) and NIL lines (IR87705-7-15-B) | Booting stage | Overall identification of 9 conserved and 34 non-conserved microRNA families as drought-responsive in rice inflorescence | Cheah et al., 2017 |
| Nagina 22,  Vandana, Pusa Basmati 1, and  IR64 | Flag leaf and  spikelets at heading stage and  roots at milky stage | I identified a group of “Cultivar-specific drought responsive” (CSDR)-miRNAs (osa-miR159f, osa-miR1871, osa-miR398b, osa-miR408-3p, osa-miR2878-5p, osa-miR528-5p and osa-miR397a). This lead to identification of the unique drought mediated dynamism and interplay of Cu and ROS homeostasis, in the flag leaves of the drought tolerant rice. | Balyan et al., 2017 |
| Common wild Rice (*Oryza*  *rufipogon* Griff.) | Vegetative stage (shoot and root samples) | Identified 200 differentially expressed miRNAs under drought stress conditions | Zhang et al., 2017 |
| Dongxiang wild rice (*O. rufipogon*, DXWR) | Vegetative stage | Identified 138 miRNAs in DXWR of which 67 novel miRNAs were signiﬁcantly affected by drought stress. In total, 200 candidate target genes were predicted and annotated for the drought stress-responsive novel miRNAs. | Fanto et al., 2018 |
| KMJ 1-12-3 | Vegetative stage | Four novel miRNAs, osa-miR12470, osa-miR12471, osa miR12472, osa-miR12473 were identified of which targets of three miRNAs could be successfully validated | Awasthi et al., 2019 |
| Pusa  Basmati 1 | Tissues from 15-day-old seedling leaves, roots, and  flag leaves | Identified 206 miRNAs across all three libraries and the regulatory network of miRNA in two different abiotic stress (high temperature and salinity | Goel et al., 2019 |
| ARC-10372 | Vegetative stage | Identified of 10 novel drought responsive miRNAs, namely, miR531, miR827, miR8175 miR977, miR6300, miR1861, miR440, miR9773, miR3982 and miR1876 which targeted most of the transcription factors such as NAC, MYB, MYC and ARF. | Singh et al., 2020 |

**Supplementary Table1:** Studies on genome-wide identification of drought responsive miRNAs in rice
