# Supplementary material for: Integration of miRNA dynamics and drought tolerant QTLs in rice reveals the role of miR2919 in drought stress response
Source: BMC Genomics. 2023 Sep 6;24:526. doi: 10.1186/s12864-023-09609-6 (PMC10481553; doi:10.1186/s12864-023-09609-6)
Supplement: Supplementary file 7 — Additional file 7. [file 12864_2023_9609_MOESM7_ESM.pptx]

## Slide 1
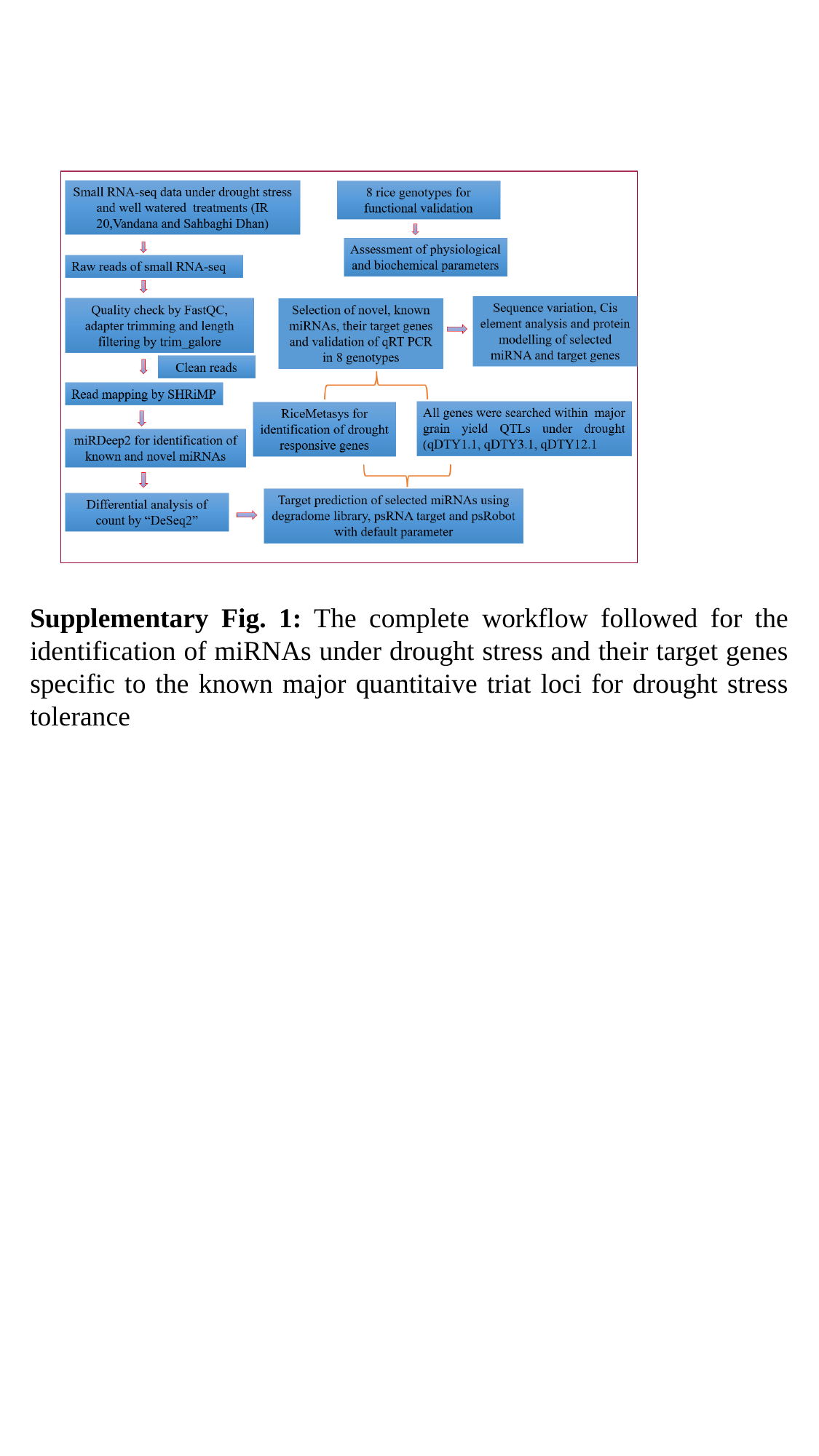

Figure 1
Supplementary Fig. 1: The complete workflow followed for the identification of miRNAs under drought stress and their target genes specific to the known major quantitaive triat loci for drought stress tolerance

## Slide 2
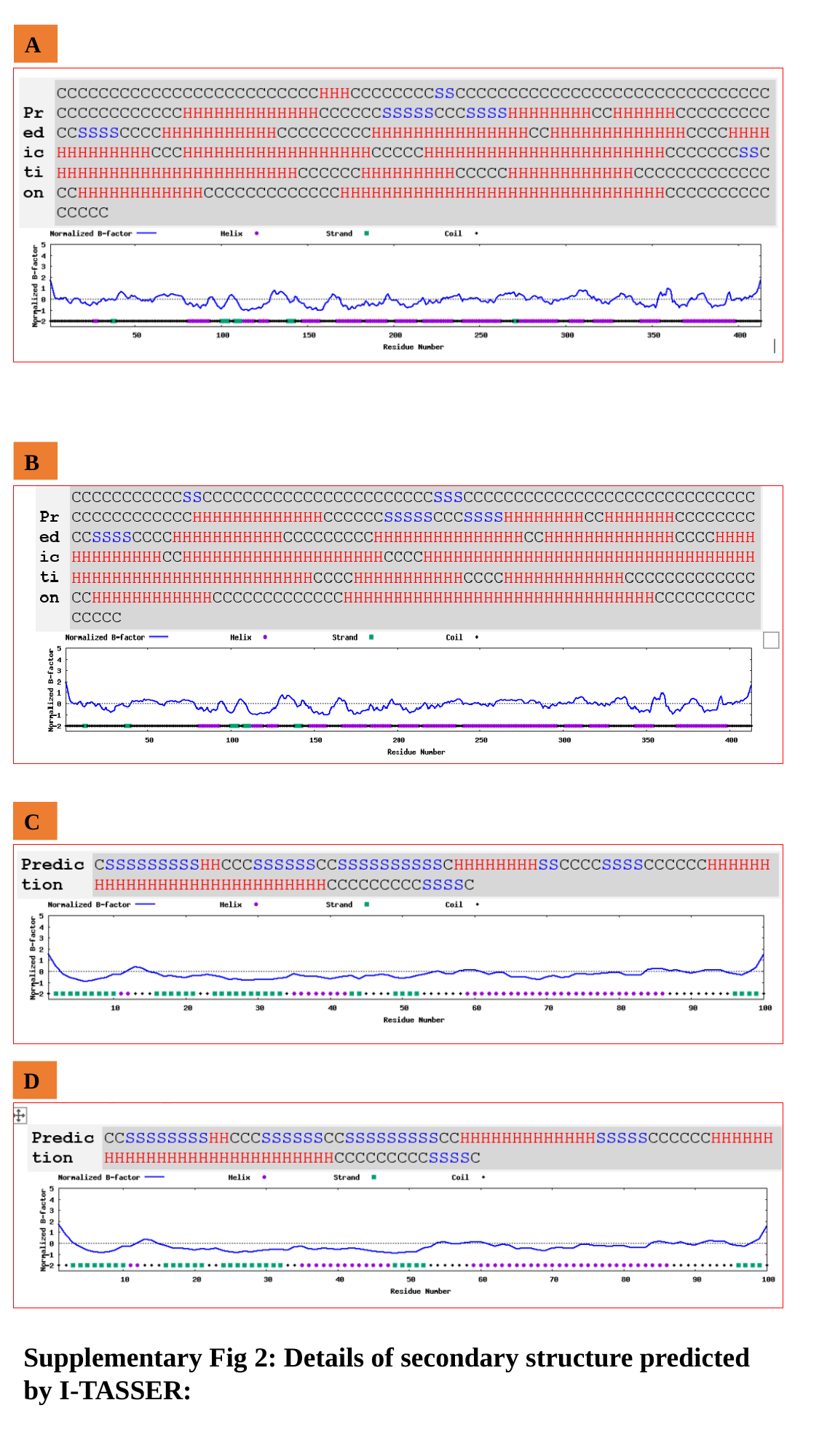

A
B
C
D
Supplementary Fig 2: Details of secondary structure predicted by I-TASSER:

## Slide 3
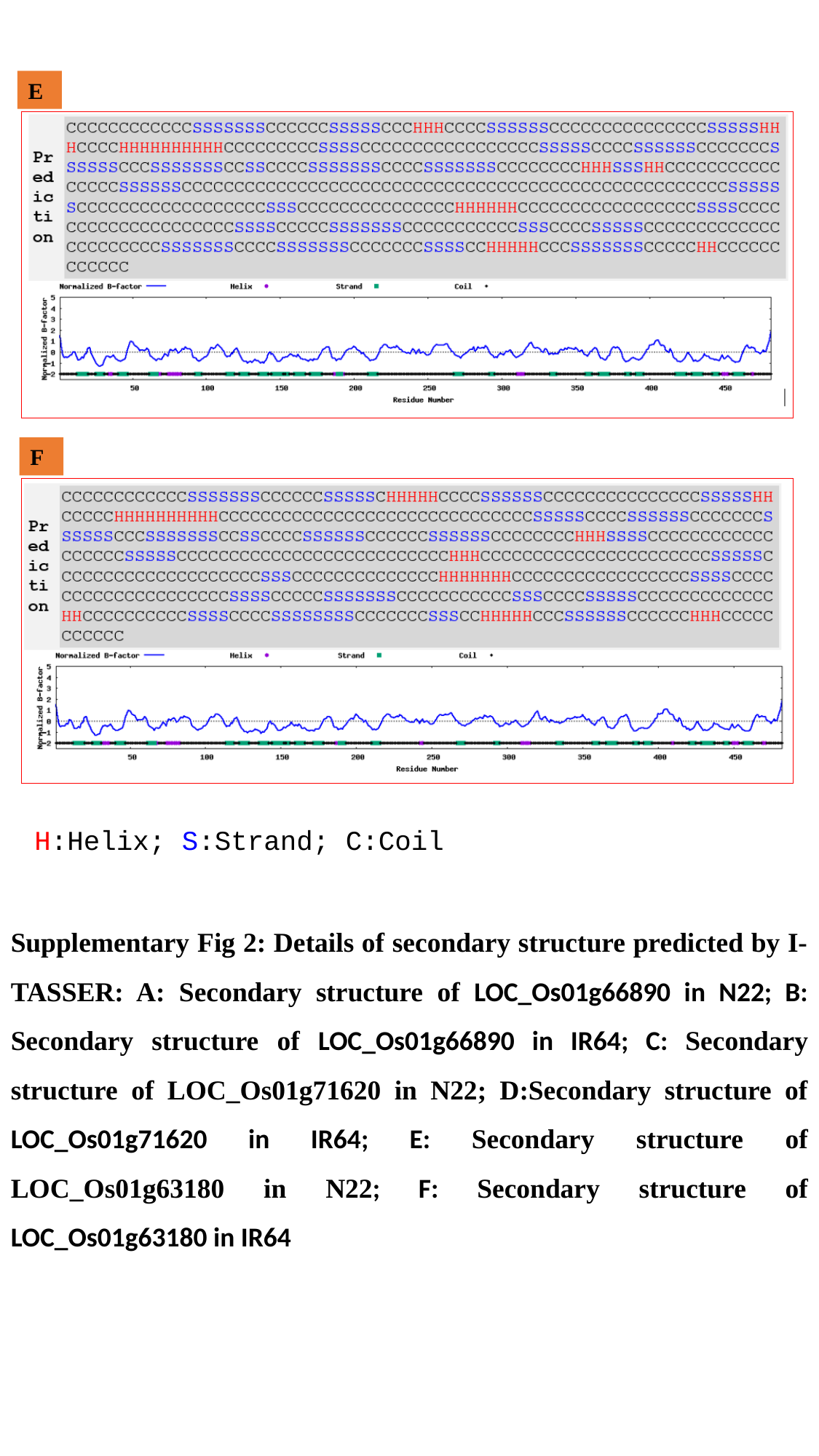

E
F
H:Helix; S:Strand; C:Coil
Supplementary Fig 2: Details of secondary structure predicted by I-TASSER: A: Secondary structure of LOC_Os01g66890 in N22; B: Secondary structure of LOC_Os01g66890 in IR64; C: Secondary structure of LOC_Os01g71620 in N22; D:Secondary structure of LOC_Os01g71620 in IR64; E: Secondary structure of LOC_Os01g63180 in N22; F: Secondary structure of LOC_Os01g63180 in IR64

## Slide 4
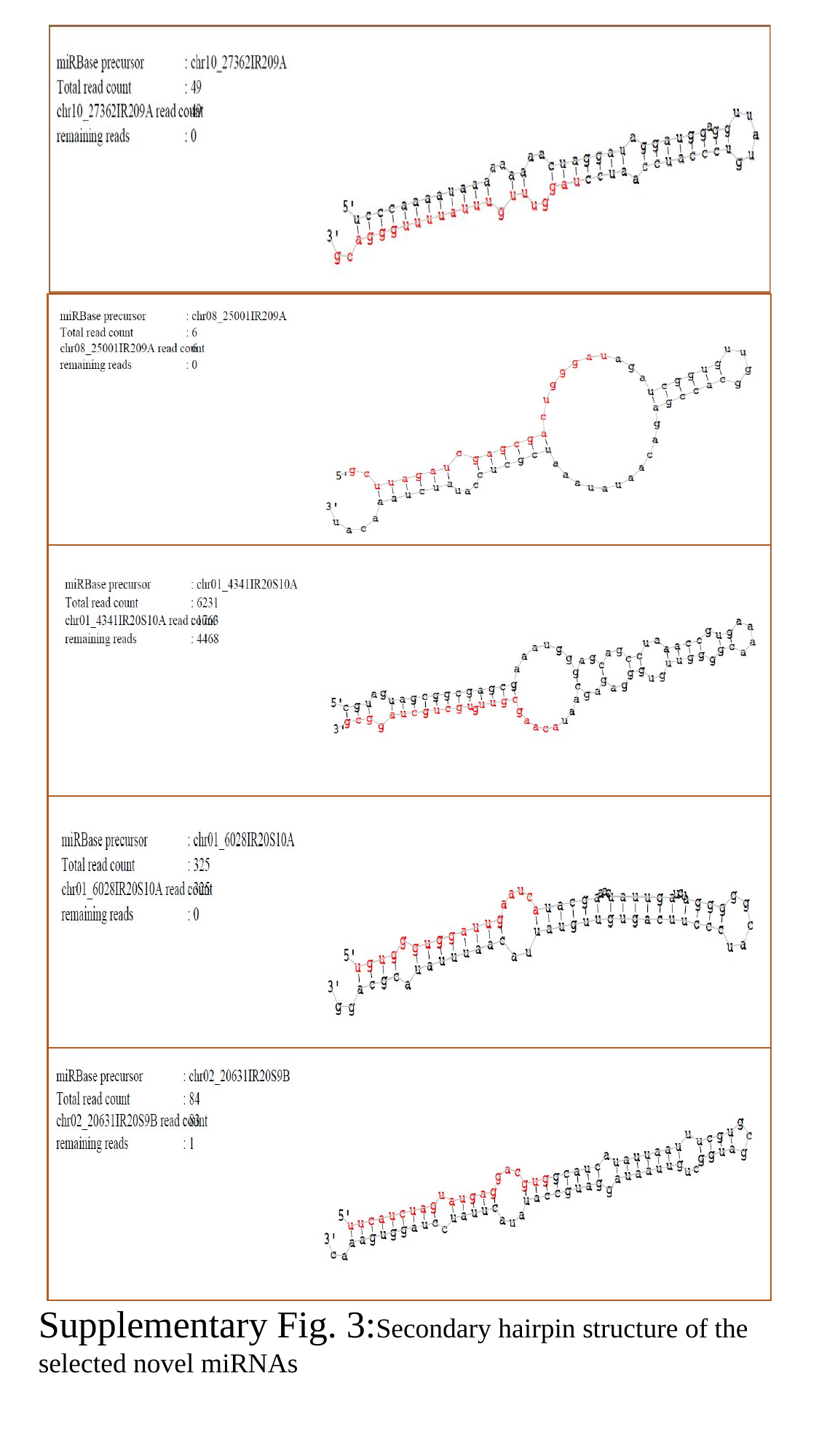

Supplementary Fig. 3:Secondary hairpin structure of the selected novel miRNAs

## Slide 5
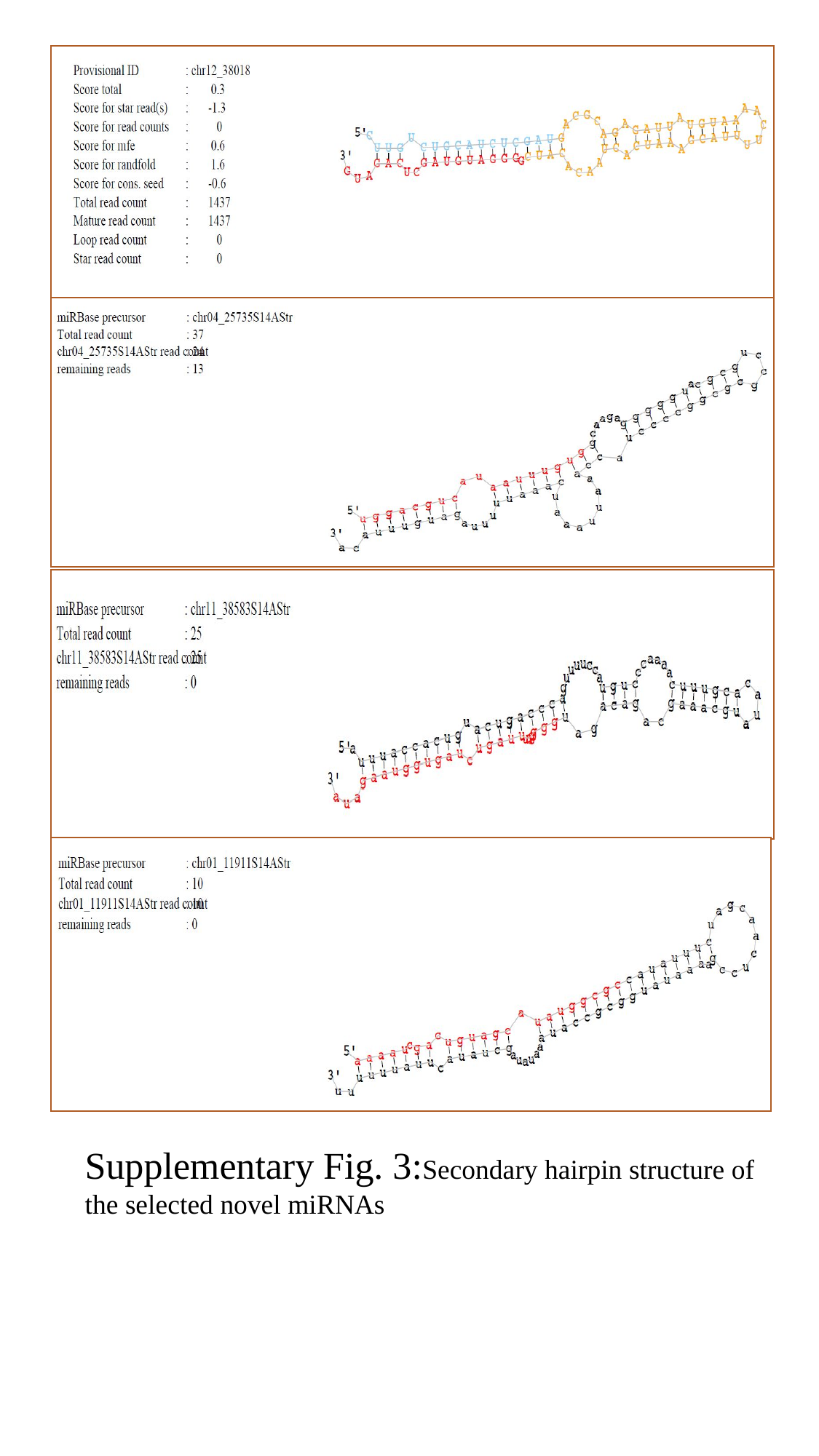

Supplementary Fig. 3:Secondary hairpin structure of the selected novel miRNAs

## Slide 6
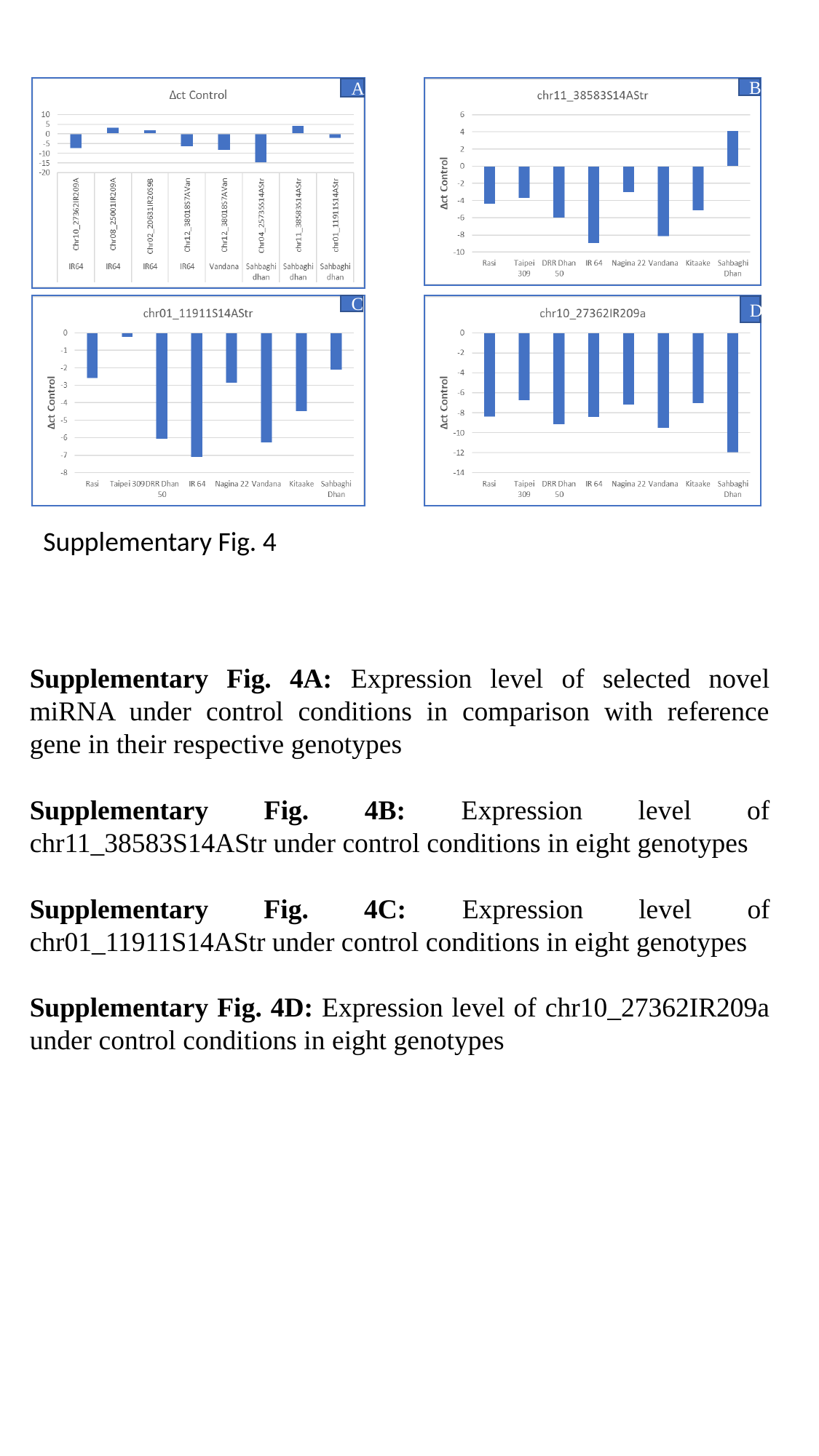

B
A
C
D
Supplementary Fig. 4
Supplementary Fig. 4A: Expression level of selected novel miRNA under control conditions in comparison with reference gene in their respective genotypes
Supplementary Fig. 4B: Expression level of chr11_38583S14AStr under control conditions in eight genotypes
Supplementary Fig. 4C: Expression level of chr01_11911S14AStr under control conditions in eight genotypes
Supplementary Fig. 4D: Expression level of chr10_27362IR209a under control conditions in eight genotypes
